# Geometry–Mechanics Compatibility Enables Interpretable Inverse Design of Non-Rigid Origami

**DOI:** 10.34133/research.1443

**Published:** 2026-09-28

**Authors:** Yucong Sun, Keyao Song, Yiqiu Wang, Wenshi Xue, Yang Li, Xiang Zhou

**Affiliations:** ^1^ School of Aeronautics and Astronautics, Shanghai Jiao Tong University, Shanghai 200240, China.; ^2^ Lab of Civil Aircraft Structures Testing, School of Aeronautics and Astronautics, Shanghai Jiao Tong University, Shanghai 200240, China.; ^3^ Aerospace Structure Research Center, School of Aeronautics and Astronautics, Shanghai Jiao Tong University, Shanghai 200240, China.; ^4^ School of Integrated Circuits, Wuhan University, Wuhan, Hubei 430072, China.; ^5^ The Institute of Technological Sciences, Wuhan University, Wuhan, Hubei 430072, China.; ^6^ Wuhan University Shenzhen Research Institute, Shenzhen, China.

## Abstract

Non-rigid origami offers a geometric route to programmable mechanical and kinematic functions in metamaterials, deployable structures, and robotic mechanisms. Yet, its inverse design remains constrained by a fundamental modeling dilemma: High-fidelity nonlinear simulations become prohibitively expensive when repeatedly applied across large design spaces, whereas reduced models are efficient but often rely on pattern-specific assumptions whose validity is difficult to assess across crease patterns. Here, we introduce a compatibility-guided inverse-design framework that uses a geometry–mechanics test to determine when a parameterized design family can be reliably represented by a single-degree-of-freedom (1-DOF) kinematic model. Once qualified, the reduced model enables rapid and interpretable exploration of the admissible design space, while incompatible cases remain evaluated using high-fidelity analysis. We demonstrate the framework by designing origami metamaterials with programmable multistability and multi-plateau mechanical responses, enabled by the identification and removal of key geometric constraints. Its broader applicability is further illustrated through target-shape morphing and task-specific robotic motion programming. By turning model reduction from an assumed approximation into an explicit design decision, this framework provides a geometry-driven route from target functions to manufacturable non-rigid origami while reducing reliance on simulation-intensive search.

## Introduction

Encoding functional behaviors into geometry is a central aspiration in architected physical systems [1–3]. Non-rigid origami provides a compelling route by allowing facets and creases to deform during motion, coupling folding kinematics with structural mechanics beyond the rigid-folding idealization [4–7]. This added compliance substantially broadens the functional design space, enabling applications across metamaterials [8–12], robotics [13–15], flexible electronics [16,17], and deployable structures [18–23]. Realizing these functionalities in a target-driven manner calls for an inverse-design framework that translates target behaviors into crease layouts.

However, inverse design of non-rigid origami remains largely simulation-centric. In current practice, nonlinear mechanical solvers are repeatedly embedded in optimization loops, or simulation-generated datasets are used to train surrogate models for rapid screening. This simulation-centric workflow has 3 recurring limitations. First, generating sufficiently rich high-fidelity data for either candidate evaluation [24] or surrogate training [25–27] (e.g., through bar-and-hinge simulations [28,29] or finite-element analysis [30–35]) is computationally expensive; this cost becomes prohibitive when screening large numbers of candidate crease layouts. Second, reduced-order models [36–40] can accelerate evaluation during optimization or surrogate-data generation, but many rely on pattern-specific kinematic or mechanical assumptions and therefore transfer poorly across crease layouts, often requiring reformulation when topology or crease layout changes. Third, when an optimization search fails to meet the target objective function, the failure is often difficult to interpret: It remains unclear whether the target is fundamentally infeasible under geometric constraints or simply lies outside the explored parameterization or design space. Collectively, these limitations point to a missing decision rule in current inverse-design workflows: a geometry–mechanics compatibility test that determines when reduced descriptions are mechanically admissible for candidate screening and when high-fidelity mechanics must remain the primary evaluator.

To operationalize this principle, we introduce a geometry-driven inverse-design framework that links a high-fidelity reference analysis to a compatibility-qualified reduced single-degree-of-freedom (1-DOF) model (Fig. 1A). In contrast to the conventional simulation-centric inverse-design workflows (Fig. 1B), our framework explicitly determines, through a geometry–mechanics compatibility test, when low-cost screening is admissible. The high-fidelity analysis resolves the nonlinear mechanical response of candidate structures using a meshless method [41–43], while the compatibility test determines whether a class of candidate designs can be represented by a 1-DOF description within a declared tolerance. Compatible cases are then screened rapidly using the reduced model, whereas incompatible cases remain in the high-fidelity analysis. This conditional reduction reduces reliance on repeated nonlinear simulations without committing to pattern-specific reduced-model assumptions, thereby improving transferability across crease layouts.

**Fig. 1.**
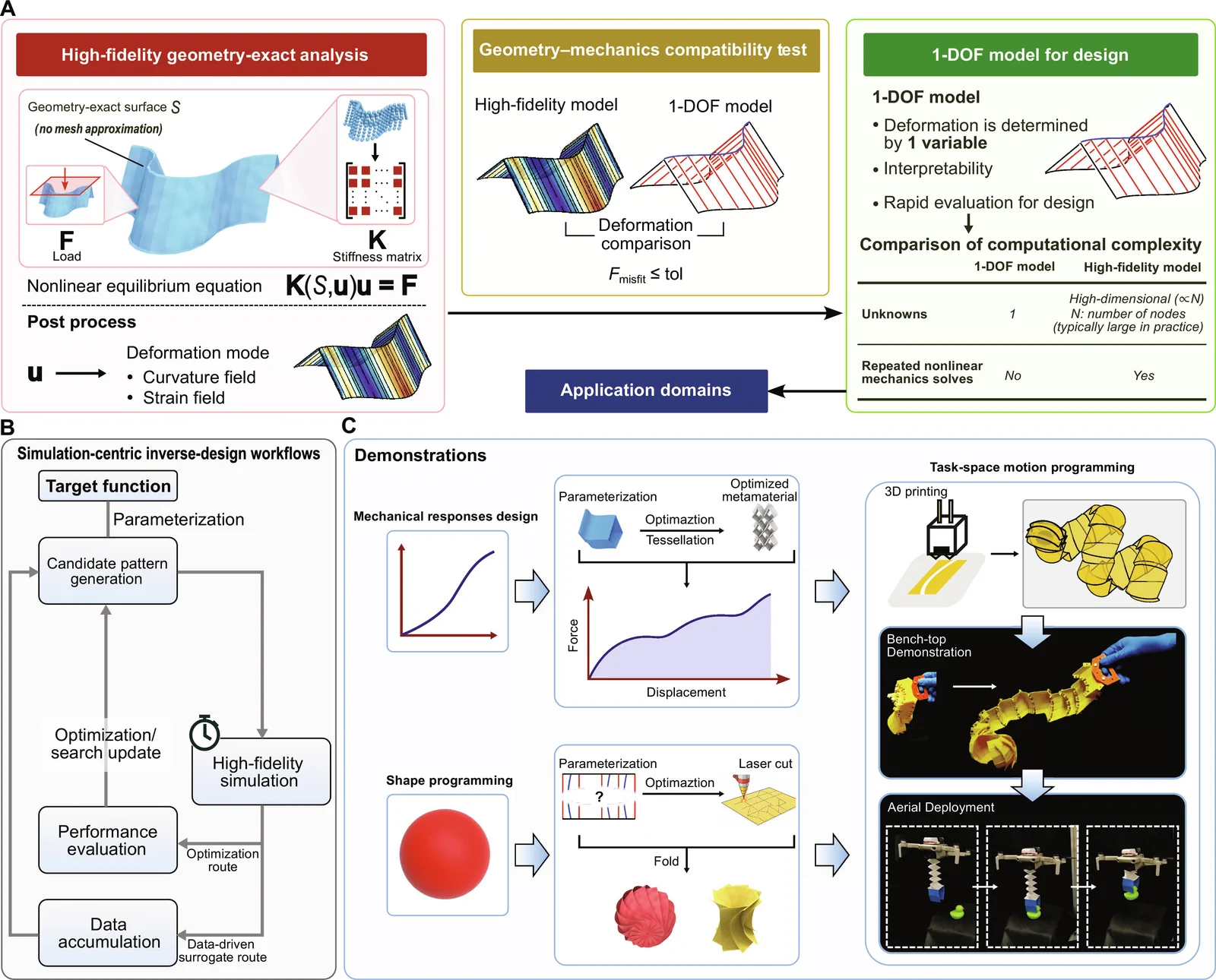
Overview of the compatibility-guided inverse-design framework for non-rigid origami. (A) Framework linking a high-fidelity geometry-exact analysis to a geometry–mechanics compatibility test and, when admissible, to a reduced 1-DOF model for rapid and interpretable design. The high-fidelity analysis solves the nonlinear equilibrium problem on the exact folded surface and outputs deformation, curvature, and strain fields. The compatibility test compares the high-fidelity response with a candidate geometric 1-DOF description and permits reduction when the normalized misfit satisfies $F_{\text{misfit}}\leq \mathrm{tol}$. For compatible cases that pass the test, the reduced model provides a low-cost evaluator for inverse design at lower computational cost in compatible cases. (B) Conventional simulation-centric inverse-design workflow, in which candidate generation, performance evaluation, optimization/search updates, and data accumulation rely on repeated high-fidelity simulations. (C) Three design domains used to test transferability across scales and functions through compatibility-qualified 1-DOF evaluation: programming mechanical responses in origami metamaterials, inverse design for target-shape morphing, and task-specific motion programming in origami robotics.

We first apply the framework to program mechanical responses in curved-crease origami metamaterials, where the reduced description reveals key geometric constraints and enables multistable and multi-plateau responses [44,45]. We further examine its broader applicability through target-shape morphing [46,47] and task-specific robotic motion programming (Fig. 1C). These demonstrations span material-, structure-, and system-level design problems and test whether the same compatibility-guided design principle remains useful across different functions and crease-pattern families. Overall, the framework turns model reduction from an assumed approximation into an explicit design decision, providing an interpretable route from target functions to realizable non-rigid origami while reducing reliance on simulation-intensive trial and error.

## Results

### High-fidelity geometry-exact analysis as the reference model

The high-fidelity reference analysis directly computes strain and curvature fields on the exact folded surface of non-rigid origami using a meshless formulation, with integration points defined on the true curved geometry rather than a mesh-approximated surface. Given an origami geometry S (facets and creases), we discretized the facets with surface nodes and assembled local node-to-node interactions within each influence domain into a global stiffness matrix $K\left(S,u\right)$, which depends on the geometry Sand the current nodal displacement field u. Boundary conditions are prescribed using the external load vector F, and the equilibrium state is obtained by solving Eq. (1):

$$K\left(S,\;u\right)u=F$$ (1)

The high-fidelity analysis yields the deformed configuration, mechanical responses along the loading path, and curvature distributions under prescribed boundary conditions. These outputs provide the high-fidelity deformation and curvature information used in the subsequent compatibility-based qualification, where geometry-consistent curvature and deformation fields are required to assess reduction admissibility. When reduction is admissible, they also serve as the reference for validating the reduced 1-DOF model.

To validate the reliability and actuation versatility of the high-fidelity reference analysis, we first considered a sheet containing a single curved crease under 3 loading modes (Fig. 2A): (a) prescribed out-of-plane edge displacement, (b) prescribed crease rotation (fold angle), and (c) prescribed panel bending. The meshless analysis captures the corresponding deformed configurations (Fig. 2B, left), while the fold-angle distributions along the normalized crease arclength agree closely with those obtained from shell-based finite element analysis (FEA) for all 3 loading modes (Fig. 2B, right).

**Fig. 2.**
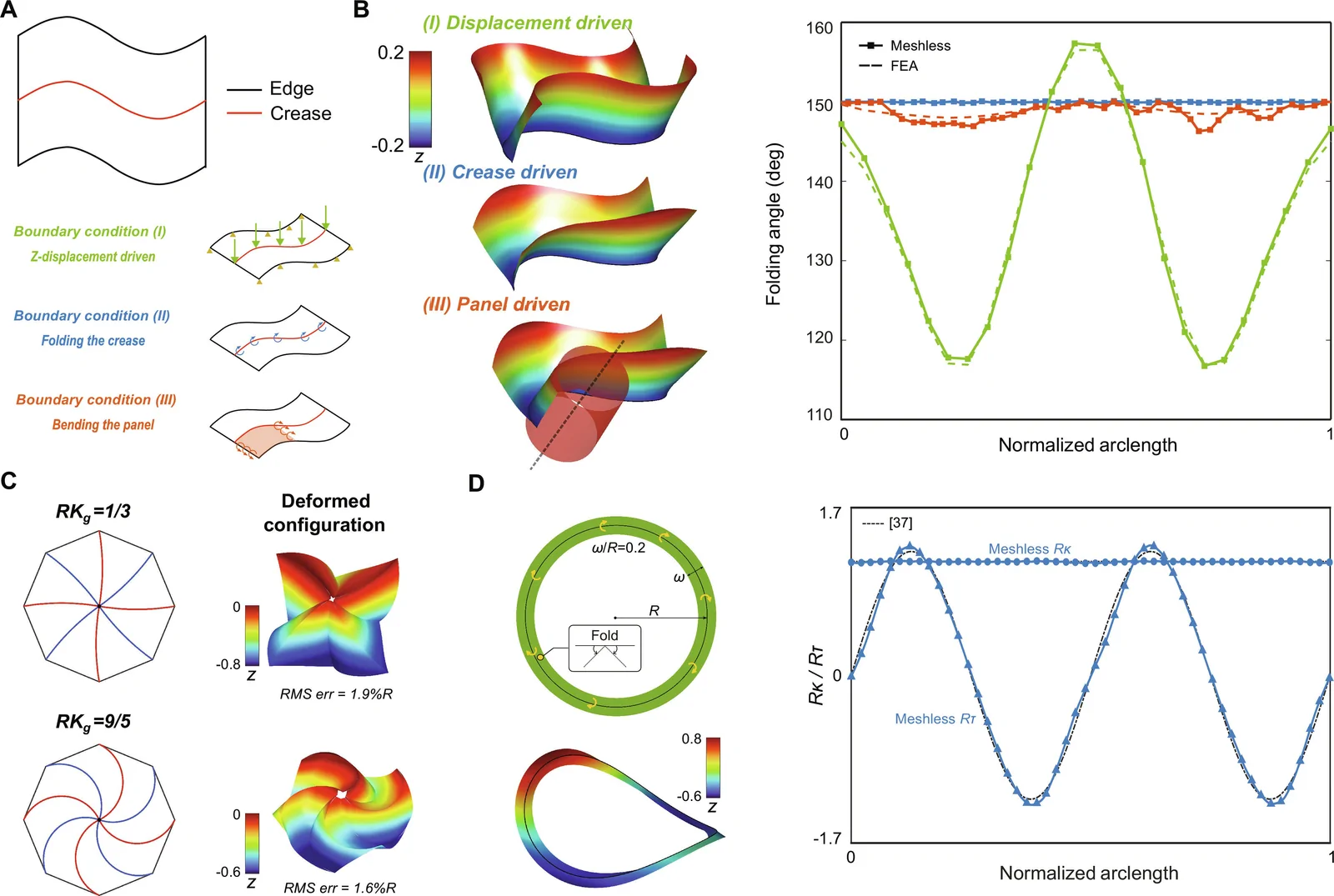
High-fidelity validation for representative origami under multiple actuation modes and topologies. (A) Geometry of a single-curved-crease sheet (black, sheet boundary; red, curved crease) and 3 loading modes: (I) prescribed displacement, (II) prescribed crease rotation (fold angle), and (III) prescribed panel bending. (B) Deformed configurations predicted by the meshless method under the 3 loading modes and the corresponding fold-angle distributions along the normalized crease arclength. (C) Multi-curved-crease configurations obtained by releasing prescribed crease rest angles, shown for ($RK_{g}=1/3$) and ($RK_{g}=9/5$), where red and blue denote mountain and valley creases, respectively. The meshless displacement fields agree closely with shell FEA, with RMS errors normalized by (*R*) of 1.9% and 1.6%, respectively. (D) Annular curved-crease pattern and its resulting buckled equilibrium configuration. The dimensionless crease curvature and torsion along the normalized arclength agree closely with the reference solution of Dias et al. [37].

To assess applicability beyond a single curved crease, we further considered multi-curved-crease and annular origami configurations. The multi-curved-crease patterns in Fig. 2C were allowed to relax from their planar states toward prescribed mountain- and valley-crease rest angles. For the 2 dimensionless geodesic curvatures of the creases considered, $RK_{g}=1/3\;$ and $RK_{g}=9/5$, the resulting meshless displacement fields agree closely with shell-based FEA, yielding *R*-normalized root-mean-square (RMS) errors of 1.9% and 1.6%, respectively. We additionally simulated the buckling of annular curved-crease origami with ω/R=0.2 (Fig. 2D). The meshless analysis reproduces the nonplanar equilibrium configuration, and the dimensionless crease curvature and torsion along the normalized arclength agree closely with the reference solution of Dias et al. [37].

Additional benchmarks covering straight-crease origami, large-scale assemblies, and further curved-crease configurations are provided in the Supplementary Materials. Together, the main-text and supplementary benchmarks establish the geometry-exact formulation as a high-fidelity reference analysis across representative origami topologies and actuation modes. We next use the deformation and curvature fields produced by this analysis to define a geometry–mechanics compatibility criterion for determining when reduction to a 1-DOF model is admissible.

### Geometry–mechanics compatibility test

We used a geometry–mechanics compatibility test to determine when a high-fidelity solution can be reliably reduced to a 1-DOF description for rapid design. The test compares the deformation field on the exact folded surface from the high-fidelity analysis with a candidate geometric 1-DOF kinematic path parameterized by a single driving folding angle θ.

From the high-fidelity analysis, we obtain the displacement field u, and the corresponding bending-strain field $\epsilon_{b}$, which measures facet deformation. In parallel, the candidate 1-DOF geometric kinematic model yields its bending-strain field $\epsilon_{b}^{\mathrm{geo}}\;$ through the curvature–folding-angle relation $\kappa \left(\theta \right)$, as illustrated by the following mapping:

$$\epsilon_{b}\leftarrow u,\epsilon_{b}^{\mathrm{geo}}\leftarrow \kappa \left(\theta \right).$$ (2)

Before comparison, the 2 bending-strain fields are mapped onto the same surface coordinates (the full form and derivation are provided in Section S3, and the mapping procedure is shown in Movie S1). We then prescreen for bending-dominated behavior using the bending-strain energy ratio of the total energy. For cases that pass this prescreen, we evaluate the agreement between the high-fidelity response and the candidate 1-DOF path using a normalized bending misfit $F_{\text{misfit}}$, and declare compatibility when

$$F_{\text{misfit}}\left(\epsilon_{b},\epsilon_{b}^{\mathrm{geo}}\;\right)\leq \mathrm{tol},$$ (3)

where tol is a prespecified admissible approximation error for design screening and should therefore be selected according to the design objective and acceptable risk, rather than prescribed as a universal constant. Recommended default ranges and a quick tuning guide for these tolerances are provided in Table S4. Cases (or candidate families) that satisfy Eq. (3) are evaluated using the reduced 1-DOF model for rapid and interpretable design, whereas noncompatible cases are retained in the high-fidelity analysis as the primary result. In this way, the compatibility test enables model reduction only when geometry and mechanics remain consistent.

### 1-DOF simplified model for rapid and interpretable design

For compatibility-qualified cases, the reduced 1-DOF model serves as the reduced-order design model, collapsing the motion to a 1-DOF description parameterized by a single driven variable. Across 4 representative benchmark cases (Fig. 3A to D and Table S3), the reduced model retains the dominant deformation mechanisms identified from the high-fidelity analysis while rewriting them in a closed form for efficient and interpretable evaluation. Specifically, the dominant mechanisms are extracted by analyzing the high-fidelity bending fields and distilled into reduced variables and coupling terms (Fig. 3E to H). Although the resulting closed-form expressions differ among the examples, they are explicit specializations of a common kinematic-energy construction. Given the prescribed geometry, ruling fields, matching constraints, and a scalar boundary value, this construction generates a candidate 1-DOF family from which the curvature and energy are evaluated; a general differential-geometric derivation for developable curved-crease families is provided in Section S4. The resulting analytical expressions reproduce the high-fidelity energy–displacement responses with close agreement in the displayed energy–displacement trends under the same loading histories (Fig. 3I to L) while replacing high-fidelity candidate evaluation with a one-parameter evaluator for compatible cases.

**Fig. 3.**
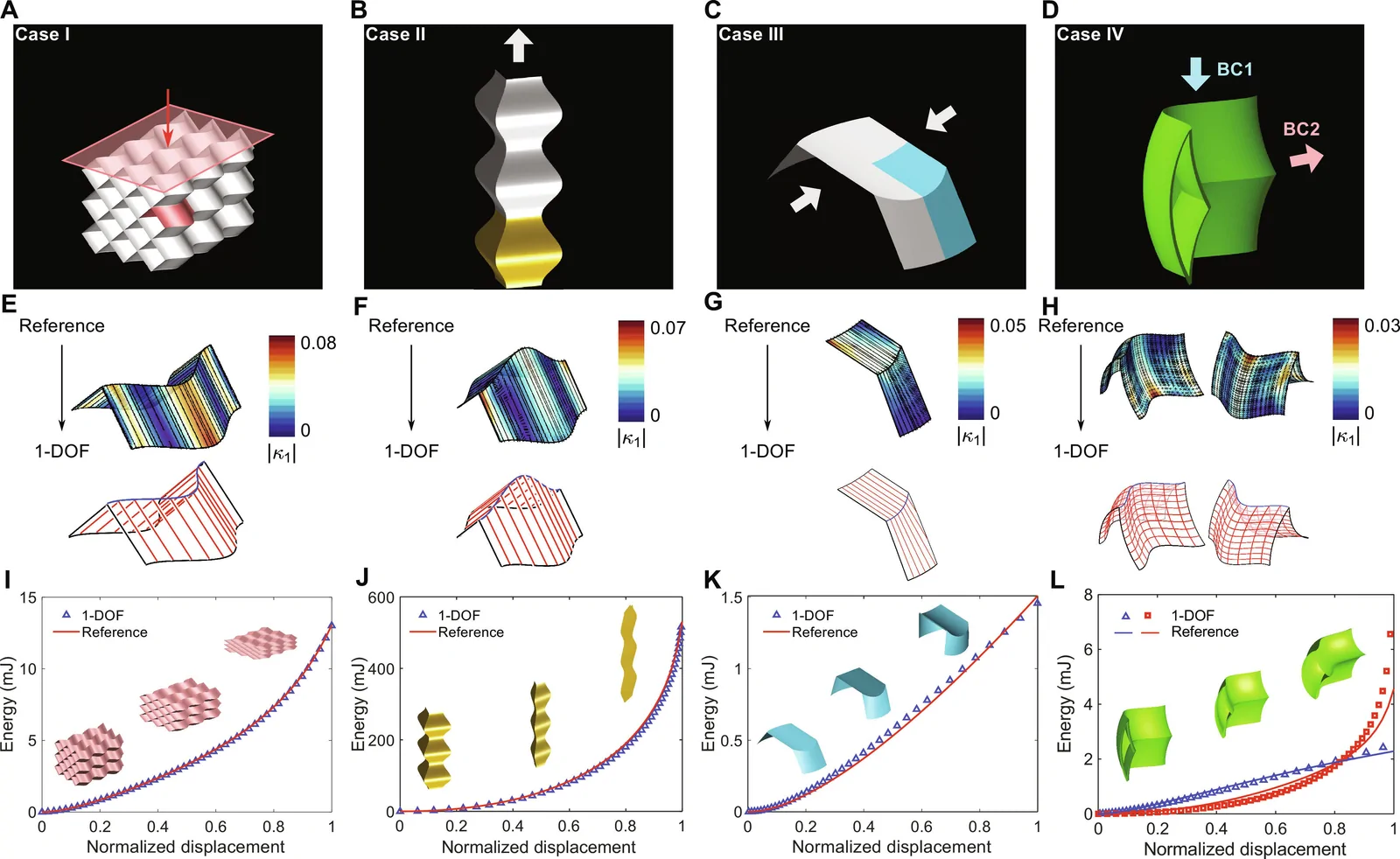
Reduced-order model informed by geometry–mechanics compatibility. (A to D) Four representative configurations and boundary conditions: (A) Euclidean origami, (B) non-Euclidean origami, (C) origami with nonparallel ruling lines, and (D) a doubly curved origami sheet (arrows denote BC1/BC2). (E to H) High-fidelity principal bending fields projected on the exact surface (top) and the distilled dominant mechanisms used to select reduced variables and coupling terms for the reduced model (bottom). (I to L) Validation under the same loading histories: Analytical predictions from the reduced model closely track the high-fidelity numerical energy–displacement responses, supporting reduced-order evaluation in compatible cases.

To illustrate how the reduced 1-DOF model is instantiated in practice, we present a closed-form geometric kinematics for the doubly curved-facet case in Fig. 3D. We parameterize the mid-surface by 2 material coordinates $\left(u,v\right)$ and a driven folding-progress variable t>0. Here,t is a single scalar monotonically related to the folding angle θ, and does not denote time. The surface geometry is encoded by 2 profile functions $f\left(u\right)$ and $g\left(v\right)$, and the folded surface $X^{t}\left(u,v\right)$ is obtained by integrating the tangent fields [Eq. (4)]. Once *t* is specified, the motion is fully determined by one scalar (1-DOF), and the resulting geometric map yields the curvature κ, which is then converted via Eq. (2) into the candidate bending-strain field used in the reduced model.

$$\begin{array}{c} X_{u}^{t}=\left(\sqrt{1+{f\prime }^{2}\left(u\right)\left(1-t^{2}\right)},0,tf^{\prime }\left(u\right)\right),\\ X_{v}^{t}=\left(0,\sqrt{1+{g\prime }^{2}\left(v\right)\left(1-t^{-2}\right)},g^{\prime }\left(v\right)t^{-1}\right). \end{array}$$ (4)

### Mechanical response programming in curved-crease origami metamaterials

We next use the framework to program mechanical response in curved-crease origami metamaterials through the reduced model. Using the double-layer curved-crease unit cell and its tessellated assembly (Fig. 4A), we compare the load–displacement responses predicted by the reduced model with those from the high-fidelity analysis and observe strong agreement at the unit-cell level (Fig. 4B), supporting the use of the reduced model for mechanical response design. With this agreement established, we then use a genetic algorithm to tune the thickness ratio of the top and bottom layers and obtain target stiffness families, including positive, near-zero, and negative stiffness responses (Fig. 4C).

**Fig. 4.**
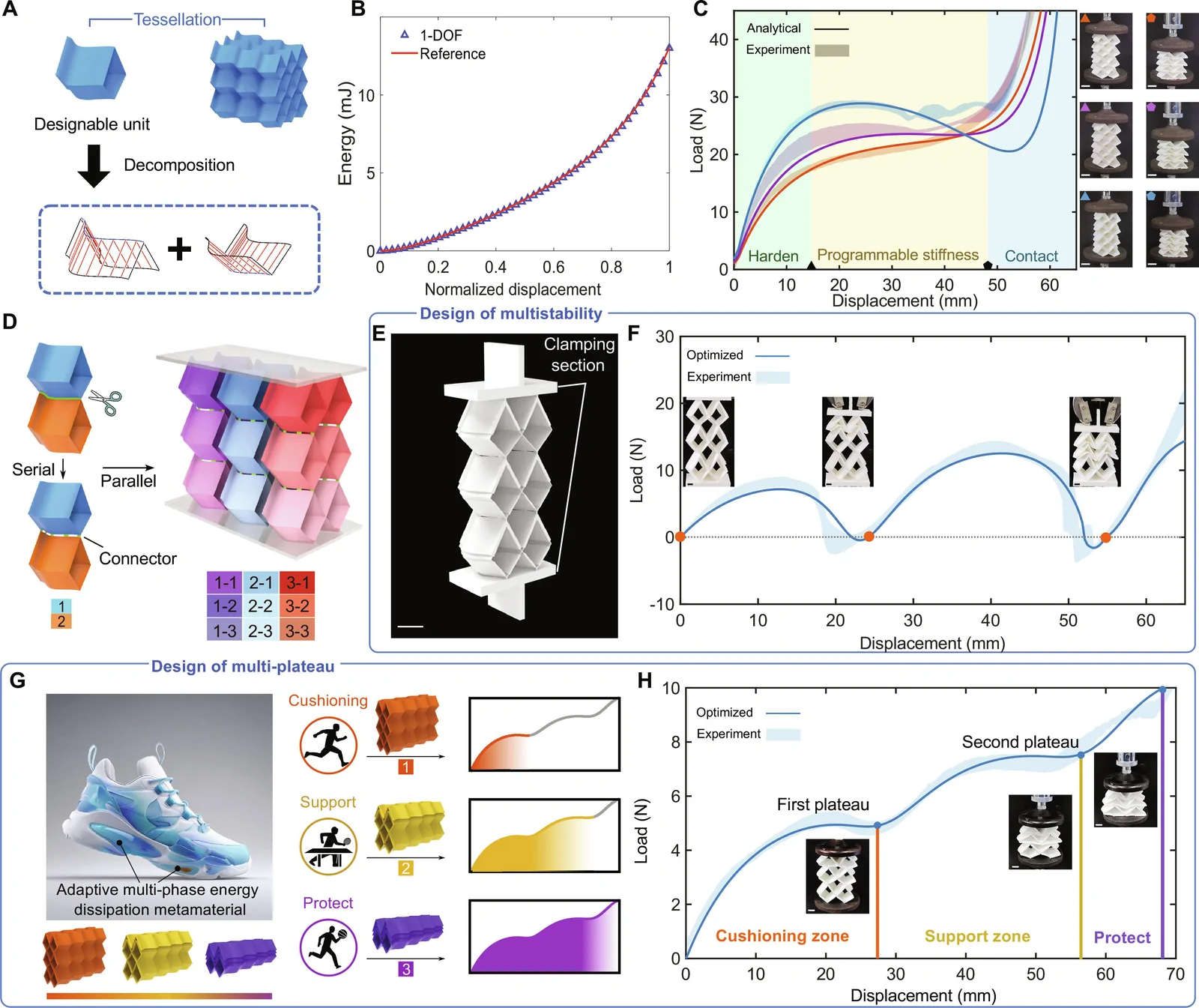
Reduced-model-based design, validation, and application of a curved-crease origami metamaterial. (A) A designable double-layer curved*-*crease unit and its 3D tessellation. (B) Comparison of the reduced model and the high-fidelity analysis under unit cell compression. (C) Families of periodic metamaterials designed to exhibit positive, near*-*zero, or negative effective stiffness by tuning layer thickness ratios; lines denote analytical designs and shaded bands show experimental envelopes across *n* = 3 independent specimens (snapshots at right). (D) Removing the green interlayer crease eliminates the dominant geometric constraint and yields a layer-decoupled serial–parallel (LSP) arrangement. (E) A clamping section enabling measurement through the force-reversal regime. (F) Multistability achieved by layer-wise design: optimized curve (line) and experimental envelope (shaded); orange markers denote stable states. (G) Insole concept using a multi-plateau energy-dissipating metamaterial: 3 segments are assigned to cushioning, support, and protection with representative single-segment plateaus (right). (H) The resulting multi-plateau load–displacement curve is partitioned into cushioning/support/protect zones; prediction tracks experiment. In (C), (F), and (H), the shaded bands represent the pointwise minimum-to-maximum load range obtained from 3 independent specimens at each imposed displacement. Scale bar, 10 mm.

Building on the interpretability of the reduced model, we identified the green interlayer crease in Fig. 4D as a kinematic constraint. With identical crease geometry ϕ, each layer experiences a crease-prescribed local compression $\delta_{j}$, while the overall motion remains series with $\delta =\sum_{j}\delta_{j}$. Under this constraint, the system energy is simply the sum of the layer energies [Eq. (5)], and the measured reaction is the derivative of the total energy with respect to δ. After cutting the crease, the layers are linked only by small connectors; they decouple into a layer-decoupled serial–parallel (LSP) system, so each layer carries the same load f [Eq. (6)]. The 2 types of energies are

$$E_{\text{total}}\left(\delta ,\phi \right)=\sum_{j=1}^{n}E^{\left(j\right)}\left(\delta_{j},\;\phi_{j}\right),\phi_{j}=\phi$$ (5)

$$E_{\text{total}}\left(\delta ,\phi \right)=\sum_{j=1}^{n}E^{\left(j\right)}\left(\delta_{j},\;\phi_{j}\right),f_{i}=f$$ (6)

where $E_{\text{total}}$ and $E^{\left(j\right)}$ represent the total energy of the serial system and each layer, respectively. $\phi_{j}$ and $f_{i}$ are the crease geometry and force of each layer.

With the kinematic constraint removed, the LSP architecture allows layer-wise optimization instead of a globally coupled search. We started from the desired load–displacement curve. Using the reduced model, we designed the first layer to match the first segment, freeze it, and then design the next layer against the remaining target; this sequential procedure continued until the full curve was met. This approach is transparent, adds layers sequentially, and benefits from the interpretability of the reduced model. To ensure reliable readings through snap-through, we added a clamping section (Fig. 4E) that kept the platens engaged when the specimen entered the negative-slope segment of the load–displacement curve. Figure 4F shows the good agreement between the optimized design and experiments (Movie S2). The curve exhibits sequential state transitions, with each orange circle marker denoting a stable state; the transitions feature force drops and a short force-reversal segment (snapshots and shaded bands indicate the configuration of the specimen and repeatability of the experiments).

Finally, as a proof-of-concept application, we embedded the designed metamaterial into an insole concept. By prescribing the activation displacement and effective stiffness of each segment, the insole delivers clearly separated plateaus that correspond to the cushioning, support, and protection zones (Fig. 4G). The overall response showed an orderly, sequential activation of these zones, and the experimental envelope tracked the optimized prediction (Fig. 4H and Movie S3). The effective crease stiffnesses and detailed layer parameters used for the designs in Fig. 4C, F, and H are provided in Tables S5 to S8. Taken together, these results show that the reduced model supports not only rapid response programming but also interpretable constraint identification and architecture-level redesign for programmable mechanical components.

### Target-shape morphing from periodic to aperiodic curved-crease patterns

We next use the framework for target-shape morphing in curved-crease origami, starting from periodic patterns and then extending to aperiodic ones. For the periodic cases (Fig. 5A to C), folding of a single crease propagates through the crease pattern via geometric compatibility, producing collective deformation with approximate rotational symmetry or enabling closed-surface formation. Using this periodic architecture, the reduced model rapidly generates candidate crease patterns for representative doubly curved target surfaces (Fig. 5C and Movie S4). Beyond fast candidate generation, the interpretability of the reduced model reveals structural constraints and design rules that improve pattern quality, as summarized below.

**Fig. 5.**
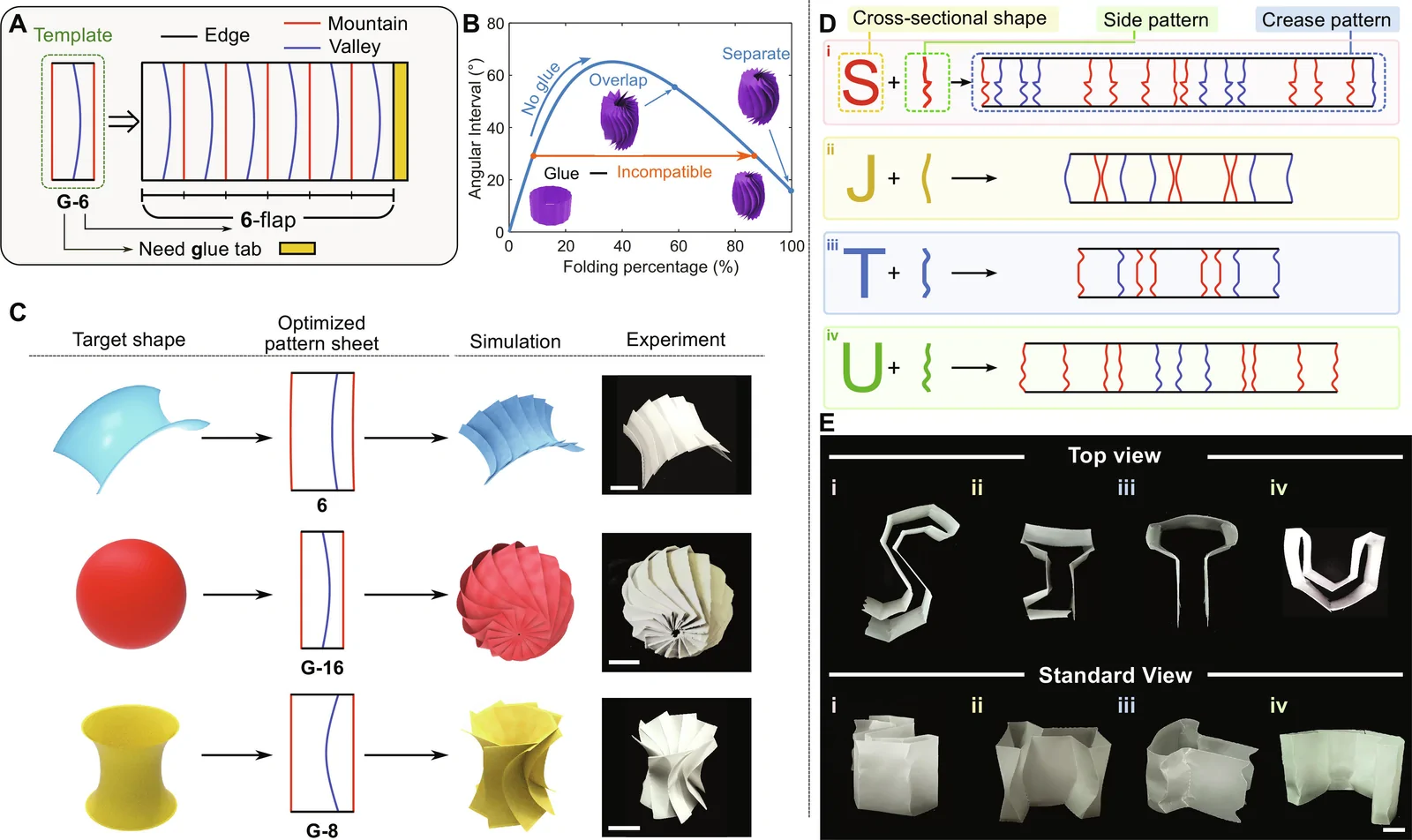
Reduced-model-based inverse design and application of curved-crease origami shapes. (A) Periodic curved-crease unit pattern and schematic decomposition. (B) Evolution of angular interval between adjacent units during folding, leading to closed configurations when boundaries are joined. (C) Inverse-designed crease diagrams and their folded models achieving representative doubly curved surfaces with optimized match to target geometries. (D) Extension from periodic to aperiodic designs: designing the targeted 3D irregular cross-section patterns and side patterns from the flat aperiodic crease patterns. (E) Examples using letter-shaped cross-sections (S, J, T, U) demonstrating reproduction of prescribed boundaries in folded models (bottom panels), shown in both top and standard views. Scale bar, 20 mm.

First, we studied a flat, curved-crease origami pattern with periodic units. In Fig. 5A, a representative periodic curved-crease pattern is shown in a simplified form. Folding any single crease initiates a rotation that propagates through the crease pattern owing to geometric compatibility, causing the entire sheet to deform collectively with approximate rotational symmetry. As folding progresses, the angular interval between adjacent facet units first increases and then decreases (Fig. 5B). When the 2 ends of the sheet are glued together, this evolution leads to a closed surface configuration. In Fig. 5C, 3 representative target surfaces with double curvature are considered together with their optimized crease patterns, and the folding process for the hourglass-like shape is shown in Movie S4. Although the low-cost reduced model inverse design framework can rapidly generate candidate patterns, the interpretability of the reduced model further reveals the structural constraints and design principles that improve the pattern quality:

1. The doubly curved shapes realized by the periodic pattern were effectively decoupled along the 2 principal curvature directions. This enables a more precise and efficient control of the final configuration by solving 2 independent nonlinear equations [Eqs. (7) and (8)].

$$F_{\text{shape}}^{\left(1\right)}\left(f_{\text{crease}},\;f_{\text{target}},\;\theta \right)=0,$$ (7)

where $f_{\text{crease}}$ and $f_{\text{target}}$ represent the flat crease function and generatrix function of the target shape, respectively. θ is the folding angle of the curved crease.

$$F_{\text{shape}}^{\left(2\right)}\left(\alpha_{\text{interval}},\;f_{\text{crease}},\;\theta \right)=0,$$ (8)

where $\alpha_{\text{interval}}$ is the angular interval between adjacent units in the target shape.

2. The feasible domain of periodic curved origami is bounded by rotational symmetry. Accordingly, we optimized the crease diagrams so that the folded structures precisely matched the desired configurations at full folding (100% folding ratio), that is, $\theta =\arctan\frac{1}{\left|f_{\text{crease}}^{\prime }\left(u\right)\right|}$.

The interpretability of the reduced model further reveals that the cross-section of an irregular target configuration must be constructed from aperiodic crease patterns, whose creases are designed to be linearly dependent. Accordingly, unlike the periodic examples in Fig. 5C, the letter-shaped examples in Fig. 5D and e were constructed directly using these model-derived geometric design rules, rather than through reduced-model-based numerical optimization. In Fig. 5D, we present crease designs that combine different cross-section patterns with irregular side patterns in a 3-dimensional (3D) configuration. For demonstration, we take the outlines of the 4 letters S, J, T, and U as the prescribed illustrative targeted cross-sectional configuration. For each case, a crease pattern is generated from the targeted cross-sectional configuration and the side patterns. Note that the prescribed cross-section configuration and the side pattern are decoupled and can be separately designed at will. The corresponding folded models (bottom panels) confirm that the sheet can accurately reproduce the desired letter-shaped boundaries in both the top and standard views. This demonstrates that the periodic-to-aperiodic generalization preserves geometric compatibility, and that the reduced model not only accelerates inverse design but also reveals structural rules for handling irregular cross-sections.

### System-level robotic function realization with modular 1-DOF origami manipulators

Having validated the proposed framework for architecture-level inverse design of programmable mechanical responses and target-shape realization, we next examine whether the same geometry-driven logic extends to system-level robotic manipulation. In this setting, the central challenge is not a single performance objective, but the concurrent realization of low control complexity, spatial programmability, fabrication accessibility, and dynamic tunability. We use a radar-chart comparison with conventional soft and continuum robots (Fig. 6A) to frame this multi-objective design problem and to motivate a modular origami route that can balance these requirements within a 1-DOF actuation scheme.

**Fig. 6.**
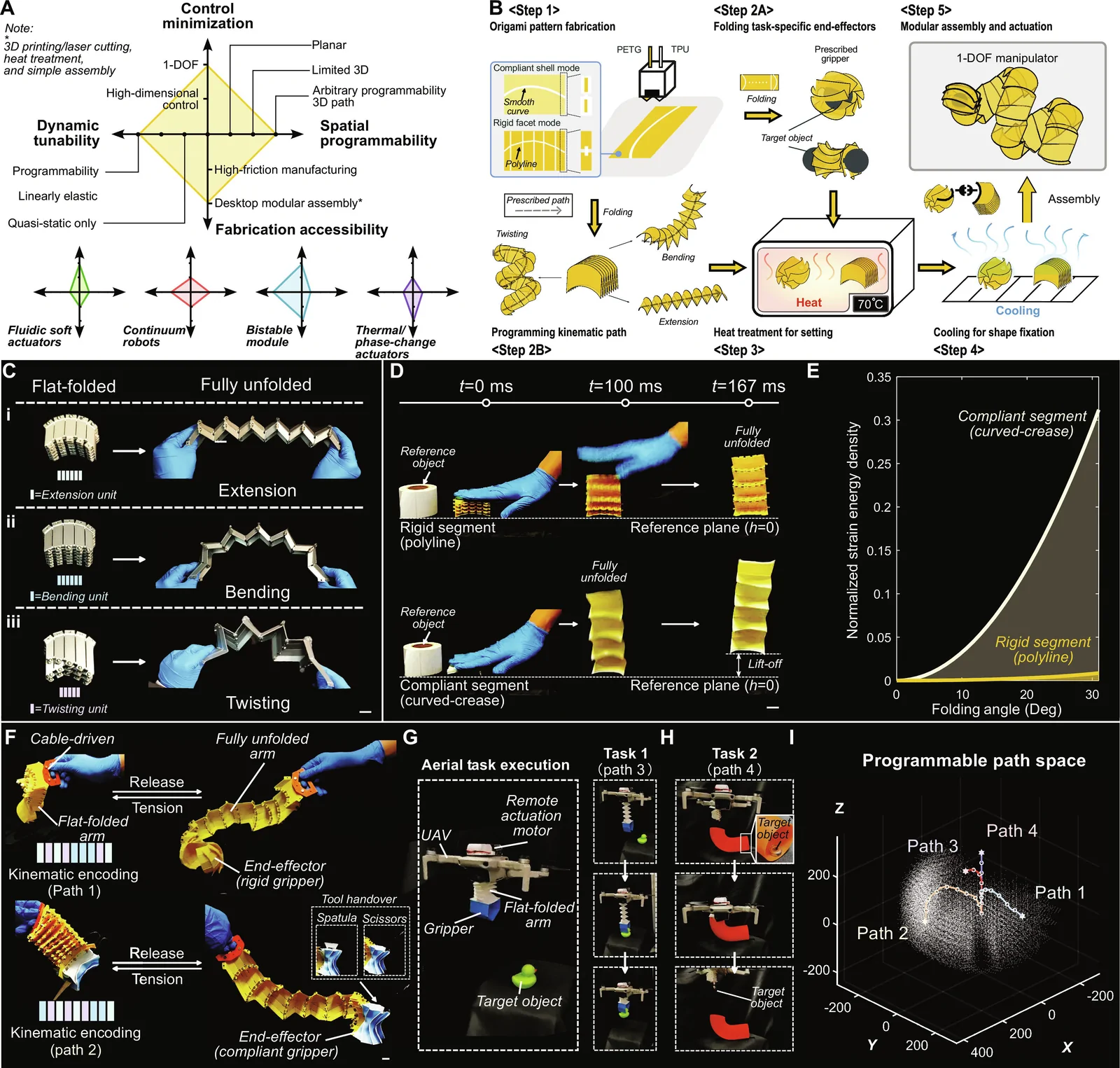
Reduced-model-based design and application of an origami manipulator. (A) Radar chart comparing the proposed manipulator with conventional soft and continuum robots, summarizing relative design trade-offs in control minimization, spatial programmability, fabrication accessibility, and dynamic tunability. (B) The streamlined “print-fold-heat-assemble” workflow, illustrating the transition from 2D composite patterns (PETG and TPU) to task-specific end-effectors and 3D kinematic modules. (C) Representative unit-level kinematic primitives encoded by different crease-pattern designs, showing extension (i), bending (ii), and twisting (iii) transitions from flat-folded to fully unfolded states. (D) Sequential snapshots comparing the dynamic deployment behaviors of rigid (polyline) and compliant (curved-crease) segments. (E) Normalized strain energy density profiles of the rigid and compliant segments, revealing the underlying elastic energy storage mechanism for rapid deployment. (F) Cable-driven modular arm demonstrations with different kinematic encodings (paths 1 and 2), yielding distinct arm morphologies and end-effector functions (e.g., rigid and compliant grippers) from a common flat-folded form. (G) Aerial task-execution setup integrating the flat-folded arm with a UAV and remote actuation motor for grasping. (H) Task demonstrations using additional programmed paths (paths 3 and 4), where path-specific arm kinematics enable different target-oriented manipulation trajectories. (I) Programmable path space reconstructed from the generated kinematic responses, with representative trajectories illustrating the accessible 3D motion envelope enabled by the modular origami design.

To operationalize this multi-objective design problem, we instantiate the framework as a modular design-to-fabrication pipeline (Fig. 6B). Starting from the prescribed task requirements, the manipulator is decomposed into 2 geometry-encoded components: a kinematic-path module, which programs the deployment trajectory, and a task-specific end-effector module, which defines the terminal interaction model and can be either co-designed using the reduced model or integrated from existing end-effectors. The modules are then realized through a unified origami fabrication workflow, in which crease patterns are fabricated as planar sheets by 3D printing (step 1), folded into programmed forms (step 2), and thermally processed to fix the encoded geometry (steps 3 and 4) before assembly (step 5). To accommodate both polyline-approximated segments and smooth curved-crease segments, we implement 2 complementary planar-forming routes within this workflow. This unified process allows trajectory generation and terminal-task specification to be developed independently and recombined in a plug-and-play manner while preserving a low-complexity 1-DOF actuation architecture for system-level manipulation. We next define a library of kinematic primitives for path encoding (Fig. 6C). By varying the crease-pattern design at the unit level, the deployed motion of each module can be programmed to realize extension, bending, or twisting, thereby providing 3 basic motion archetypes from a common flat-folded starting state (Movie S5). In this way, a kinematic path is not designed as a monolithic geometry, but synthesized as a sequence of discrete origami units with prescribed primitive motions. This modular representation converts 3D path generation into a combinatorial design problem: By arranging and combining extension-, bending- and twisting-type units, a wide range of spatial trajectories can be constructed within the same fabrication and actuation architecture. For example, using 3 extension units, 3 bending units, and 3 twisting units, their permutations already yield 49,564 candidate path configurations, illustrating the richness of the accessible path space even with a compact primitive set.

Beyond path encoding, robotic tasks may also benefit from controllable release behavior. Although the present framework is quasi-static, it predicts the geometry-dependent elastic energy stored along the folding path, which provides an energetic design variable for influencing the subsequent deployment response. Here, we illustrate this tuning axis using 2 representative implementations of the same kinematic segment design, namely, a polyline-based (rigid-segment) version and a curved-crease (compliant-segment) version (Fig. 6D and E). Time-resolved deployment snapshots (Fig. 6D) show qualitatively distinct responses under the same actuation scenario: While both segments achieve the intended unfolding motion, the curved-crease segment exhibits a more pronounced release-driven response, including stronger out-of-plane lift-off near full deployment (Movie S5). These results indicate that dynamic response can be tuned without changing the overall modular architecture or 1-DOF actuation scheme. The energetic basis for this behavior is shown in Fig. 6E, which compares the normalized strain-energy density as a function of folding angle for the 2 segments’ realizations. The curved-crease segment stores substantially more elastic energy over the same folding-angle range than the polyline segment, providing a plausible mechanistic explanation for its stronger dynamic release during unfolding. The present model therefore provides a quasi-static energetic interpretation rather than a quantitative prediction of deployment time, velocity, or acceleration, which also depend on inertia, damping, actuation and release conditions, and rate-dependent material effects. This higher energy-storage density (and corresponding storage efficiency per structural volume) makes curved-crease segments potentially useful when tasks require more pronounced or rapid deployment responses, whereas polyline realizations remain useful when a more restrained response is preferred. Although illustrated here with polyline and curved-crease examples, the same dynamic-tuning principle is more general and can be extended through broader crease-geometry parameterization.

Guided by the primitive-based path encoding and segment-level dynamic tuning described above, we next assemble system-level manipulators by combining kinematic-path modules with interchangeable end-effectors under a common cable-driven 1-DOF architecture (Fig. 6F and Movie S6). Using the same units and actuation scheme, different kinematic encodings (e.g., paths 1 and 2) generate distinct deployed arm morphologies, while terminal functions can be switched through different end-effectors, including rigid and compliant grippers. This modular recombination enables rapid reconfiguration of manipulator embodiments without redesigning the full system. We then demonstrate deployment in an aerial manipulation setting (Fig. 6G). The left panel shows the integration of the flat-folded origami arm onto a unmanned aerial vehicle (UAV) with a remote actuation motor, highlighting compact packaging before deployment (Movie S6). The right panel shows a representative grasping task (task 1), in which the deployed arm reaches and manipulates a target object under remote actuation. A second task (task 2) further evaluates path-specific manipulation in a more constrained environment, where the encoded arm trajectory is used to approach and grasp an object located inside a pipe-like channel (Fig. 6H). These demonstrations show that the same modular pipeline supports both open-space and constrained-space manipulation by selecting task-matched path encodings and end-effectors. The 4 demonstrated trajectories (paths 1 to 4) are summarized in the programmable path-space map (Fig. 6I), illustrating that these task demonstrations are representative samples within a broader accessible 3D motion envelope.

Together, these results show that the proposed framework extends beyond origami pattern generation to system-level robotic function realization through a common geometry-driven design logic. By decoupling path encoding, dynamic response shaping, and end-effector selection within a modular 1-DOF architecture, the framework enables task-oriented manipulator reconfiguration without redesigning the entire system. The aerial and constrained-space grasping demonstrations further illustrate its feasibility in scenarios where compact packaging and remote actuation are required. More broadly, this robotic application supports the transferability of our inverse-design framework from programmable mechanical and geometric functions to task-specific motion and manipulation.

## Discussion

This work proposes a compatibility-guided inverse-design framework that links geometry-exact analysis to rapid, interpretable design for non-rigid origami. Our central contribution is not simply a faster inverse-design workflow, but a compatibility-guided validity principle for model reduction in non-rigid origami. The key challenge in this class of problems is not only how to accelerate candidate evaluation but also how to determine when a reduced description remains reliable and when high-fidelity mechanics must remain the primary evaluator. In this work, the geometry–mechanics compatibility test serves as an operational criterion for that decision, bridging geometry-exact analysis and reduced-order inverse design within a single workflow. Within the broader landscape of compliant-origami modeling, Zhang et al. [48] recently introduced a geometry-consistent discrete differential geometry (DDG) framework that represents panel elasticity and crease rotation within a unified variational formulation and further extends to nonlinear stability and robot-scale dynamics. The present study addresses a complementary question at the level of model hierarchy, rather than proposing an alternative numerical discretization: Given a high-fidelity mechanical response, when may it be represented, within a prescribed tolerance, by a low-dimensional model for repeated inverse design? Thus, the DDG framework broadens the physical behaviors accessible to unified forward simulation, whereas the present compatibility-guided framework determines when high-fidelity analysis can be replaced by an interpretable and computationally efficient 1-DOF design model.

The results demonstrate that this compatibility-guided design logic can be applied across 3 distinct classes of function-driven inverse-design problems. In mechanical metamaterials, the reduced representation supports response programming while exposing hidden kinematic constraints, enabling an architecture-level redesign rather than parameter tuning alone. In target-shape design, the same framework extends from periodic curved-crease patterns to aperiodic constructions, where interpretability helps identify feasibility boundaries and motivates structural extensions for irregular target cross-sections. In robotics, the framework extends further to system-level function realization, where path encoding, stored-energy tailoring, and end-effector selection are organized within a modular 1-DOF architecture. Taken together, these demonstrations support a claim of transferability across these design settings not because one reduced model fits all patterns, but because the workflow explicitly qualifies when reduction is valid.

At the same time, the framework has a defined scope. Its performance depends on the quality of the high-fidelity reference fields and on the suitability of a dominant low-dimensional deformation description for the candidate family under consideration. The compatibility criterion is therefore best viewed as an admissibility check for reduction, not as a substitute for high-fidelity mechanics. Cases that fail compatibility, or cases whose behavior is not well captured by a dominant 1-DOF mode, should remain in the high-fidelity analysis. In addition, practical use of the framework will depend on how transparently the compatibility threshold is selected and reported across geometries, loading conditions, and design objectives. These considerations do not weaken the framework; rather, they define the conditions under which compatibility-guided reduction can be used reliably and interpretably.

The current version of the geometry–mechanics compatibility test focuses on situations in which the origami facets mainly bend. It is not designed to handle cases with a strong coupling between bending and stretching or where self-contact between facets is the dominant factor. In such cases, we used the full high-fidelity analysis as the final result and did not use the simplified model. To extend our framework to these more complex situations, we will need to improve the initial screening criteria and allow for a more advanced simplified model that can account for stretching. We believe that the same overall workflow (high-fidelity analysis → compatibility test → reduced model) will remain effective. Two directions appear promising for future research. First, adapting both the high-fidelity analysis and the reduced model to handle dynamic, time-dependent problems would allow us to study transient behaviors, the onset of instability, and rate-dependent effects in deployable or robotic systems. Second, improving the high-fidelity model to explicitly handle self-contact and expanding the reduced model to include the effects of stretching would substantially broaden the range of problems that our framework can solve.

## Materials and Methods

### High-fidelity modeling of non-rigid origami

The high-fidelity model handles both straight- and curved-crease origami within a unified geometry-exact framework. The 2 classes differ only at the input preprocessing stage, whereas the facet and crease formulations and quasi-static solving are identical. Input preprocessing is summarized in Section S2.1; facet and crease formulations are provided in Sections S2.2 to S2.4; and solver settings are presented in Section S2.5. A consolidated notation list is provided in Table S1, while the overall mode I workflow and numerical integration strategy are illustrated in Figs. S1 and S2, respectively. The representative configurations and loading modes are illustrated in Fig. 2. The detailed boundary conditions for Fig. 2 and additional high-fidelity results are provided in Table S2 and Section S7.3.

### Normalized misfit for the compatibility test

The step-by-step construction of the geometry–mechanics compatibility test is provided in Section S3. Here, we only define the normalized misfit $F_{\text{misfit}}$ used in Eq. (3) in the energy-norm form, as shown in Eq. (9).

$$F_{\text{misfit}}\left(\epsilon_{b},\epsilon_{b}^{\mathrm{geo}}\right)={\left(\frac{\int_{\Omega }{\left(\epsilon_{b}-\epsilon_{b}^{\mathrm{geo}}\right)}^{T}D_{b}\left(\epsilon_{b}-\epsilon_{b}^{\mathrm{geo}}\right)dA}{\int_{\Omega }{\left(\epsilon_{b}\right)}^{T}D_{b}\left(\epsilon_{b}\right)dA+\delta_{b}}\right)}^{\frac{1}{2}}$$ (9)

where $\epsilon_{b}$ and $\epsilon_{b}^{\mathrm{geo}}$ denote the bending strain field on the facets, which is the bending strain vector at Gauss points. $D_{b}$ is the bending constitutive sub-block. The small constant $\delta_{b}$ is a numerical safeguard (fixed at 10^−10^) to avoid division by a near-zero denominator. This normalization yields a dimensionless, scale-insensitive measure; expanded expressions and implementation details are provided in Section S3.

### Reduced 1-DOF kinematic model

In this study, we adopted a 1-DOF kinematic model as the reduced model. For cases that pass the geometry–mechanics compatibility test, facets evolve through quasi-isometric deformations during folding. We parameterize the motion by a single driven variable (e.g., a representative crease angle θ or a path parameter *t*) and recover the configuration $X^{t}$ by constraining each facet to remain isometric to its initial metric (pointwise). The explicit construction, including the closed-form relation in Eq. (4), and derivation are provided in Section S4. Once the kinematic path was identified, the mechanical responses were obtained by evaluating the energy along the driven variable, yielding the force–displacement curves reported in the Results section.

### Inverse design with the reduced model

We cast the inverse design as a constrained optimization problem over the design parameters p (crease layout/geometry, thickness, etc.). Because the reduced 1-DOF model has low computational cost after compatibility qualification, it can be coupled with both gradient-based solvers and heuristic searches [Eq. (10)].

$$\min_{p\in P}J\left(p\right)\;s.t.\;\mathrm{Ceq}\left(p\right)=0,C\left(p\right)\leq 0$$ (10)

where J is the objective function, which is used to measure the difference between the optimized and expected properties. $C\left(p\right)$ collects the geometry bounds. Ceq and C respectively represent equality constraints and inequality constraints in the optimization process. Equality constraints may include the requirements that the optimized structure must meet at certain specific positions, whereas inequality constraints may include the range of motion of the optimized structure. Implementation details for the inverse-design formulations are provided in Section S5.

### Fabrication and experiments

All metamaterials were printed monolithically using direct 3D printing. Creases were realized as locally thinned “living hinges” of prescribed width, yielding a line spring whose effective torsional stiffness per unit length scales with the third power of the hinge thickness (nominal values from thin-plate theory), and was fine-tuned by local thickness control. For each design, we tested *n* = 3 independent specimens under identical boundary conditions and displacement-controlled quasi-static loading. The setup of the universal testing machine is shown in Fig. S7.

## Data Availability

All data are available in the main text or the Supplementary Materials.

## Supplementary Materials

Supplementary 1 Sections S1 to S8 Figs. S1 to S11 Tables S1 to S8 Movies S1 to S6 Data S1 and S2
