# Supplementary material for: Geometry–Mechanics Compatibility Enables Interpretable Inverse Design of Non-Rigid Origami
Source: Research (Wash D C). 2026 Sep 28;9:1443. doi: 10.34133/research.1443 (PMC13617216; doi:10.34133/research.1443)
Supplement: Supplementary file 1 [file research.1443.f1.zip › SM_revision_unmarked.pdf]

Supplementary Materials

Geometry–Mechanics Compatibility Enables Interpretable Inverse Design of Non-Rigid Origami

Yucong Sun[†], Keyao Song[†], Yiqiu Wang, Wenshi Xue, Yang Li, Xiang Zhou[*]

[*]Corresponding author. Email: xiangzhou@sjtu.edu.cn; Tel: +86-21-34207538

[†]These authors contributed equally to this work.

This Supplementary Material includes:

Sections 1–8

Figures S1 to S11

Tables S1 to S8

Captions for Movies S1 to S6

Captions for Data S1 to S2

Additional Supplementary Materials files:

Movies S1 to S6

Data S1 to S2

Supplementary Material

1 Notation and Conventions

This section summarizes the symbols, coordinate frames, and sign conventions used throughout the Supplementary Materials. A consolidated notation table is provided later in Table S1 so that all tabulated material appears together under the Tables section.

2 Meshless Analysis of Non-Rigid Origami (Mode I)

Mode I is our geometry-exact meshless analysis for non-rigid origami. We model facets that can undergo large rotations together with membrane, bending, and transverse-shear strains. Facets are represented by an **adapted Reissner–Mindlin shell** with rotation updates via the Rodrigues formulation, and creases are treated as kinematic interfaces that can be free hinges, torsional springs with a rest angle, or angle-locking constraints. Self-contact is neglected unless otherwise stated. The main procedure is illustrated in Fig. S1.

2.1 Input Origami

Straight-crease origami A straight-crease origami is defined by a planar graph $(\mathcal{V}, \mathcal{E}, \mathcal{F})$: vertex coordinates V in a right-handed global frame; crease edges $\mathcal{E} \subset \mathcal{V} \times \mathcal{V}$; and faces $\mathcal{F}$ given by **clockwise** vertex indices to fix the outwards normal. Each crease carries attributes: initial folding angle θ_0, and torsional stiffness k_θ (per unit length).

Curved-crease origami Curved-crease origami is defined by multiple surfaces $S(u, v)$ over closed parameter domains, together with crease curves $\Gamma_c : s \mapsto \mathbf{x}(s) \subset S$. We sample Γ_c by arc length and attach a local frame $(\mathbf{t}, \mathbf{n}, \mathbf{b})$ for curvature and angle definitions.

2.2 Facet Modelling

The RKPM method in the meshless approach is adopted to model the facet of non-rigid origami.

Discretization. Facets are discretised into nodes $\mathbf{x}_I$ and integration points $\mathbf{x}^h$ on the **geometry-exact** surfaces. Each node has six DOFs (three translational DOFs + three rotational DOFs). The shape functions Ψ_I are used to interpolate the field at the integration points.

$$\mathbf{u^h}(\xi, \eta) = \sum_{I=1}^{NP} \Psi_I \mathbf{u}_I \tag{S1}$$

where (ξ, η) represents the parameter of the origami facet. The shape function is derived through Eqn. (S2):

$$\begin{aligned}
&\Psi_I(\mathbf{x}) = H^T(\mathbf{x} - \mathbf{x}_I)c(\mathbf{x})\phi_a(\mathbf{x} - \mathbf{x}_I), \\
&H(\mathbf{x} - \mathbf{x}_I) = [1, x - x_I, y - y_I, (x - x_I)^2, \ldots, (x - x_I)^n]^T, \\
&M(\mathbf{x}) = \sum_{I=1}^{NP} H(\mathbf{x} - \mathbf{x}_I)H^T(\mathbf{x} - \mathbf{x}_I)\phi_a(\mathbf{x} - \mathbf{x}_I), \\
&c(\mathbf{x}) = M^{-1}(\mathbf{x})H(\mathbf{0}).
\end{aligned} \tag{S2}$$

Finally, the complete form of the shape function is expressed as

$$\Psi_I(\mathbf{x}) = H^T(\mathbf{0})M^{-1}(\mathbf{x})H(\mathbf{x} - \mathbf{x}_I)\phi_a(\mathbf{x} - \mathbf{x}_I). \tag{S3}$$

Kinematics of Adapted Reissner-Mindlin Shell. Next, we specify the Reissner–Mindlin shell kinematics used to evaluate strains and rotations. In a three-dimensional shell (a curved structure with a finite thickness), the position of any point within the shell can be uniquely represented by a point on the mid-surface, the unit normal vector at that location, and the signed distance from the mid-surface along the normal direction.

$$\mathbf{x^0} = \mathbf{x^0_m}(\xi, \eta) + \zeta \mathbf{n^0}(\xi, \eta) \tag{S4}$$

where ζ denotes the signed distance along the unit normal vector $\mathbf{n^0}$, $\mathbf{x^0}$ and $\mathbf{x^0_m}$ represent the position of a point in the shell and its projection onto the mid-surface in the initial configuration, respectively. The superscript $(\cdot)^{\mathbf{0}}$ indicates quantities in the initial configuration, whereas the subscript $(\cdot)_{\mathbf{m}}$ refers to the mid-surface.

Given that facets in origami structures may undergo large rotations and deformations, this study adopts the large-deflection shell theory with an adapted thickness direction. The position vector of a point in the deformed three-dimensional shell is expressed as

$$\mathbf{x^t} = \mathbf{x_m^t}(\xi, \eta) + \zeta \mathbf{n^t}(\xi, \eta) \tag{S5}$$

where $\mathbf{n^t}$ denotes the unit normal vector to the deformed mid-surface, which is obtained through the rotational transformation of the initial thickness direction vector, expressed as $\mathbf{n^t} = \mathbf{R^t n^0}$. The rotation matrix is derived using Rodrigues formula (S6).

$$\mathbf{R^t} = \exp(\mathbf{S}) = \mathbf{I} + \frac{\sin(\omega)}{\omega}\mathbf{S} + \frac{1 - \cos(\omega)}{\omega^2}\mathbf{S}^2 \in SO(3) \tag{S6}$$

where

$$\mathbf{S} = \begin{bmatrix} 0 & -\phi & \theta \\ \phi & 0 & \psi \\ -\theta & -\psi & 0 \end{bmatrix} \tag{S7}$$

and $\omega = \sqrt{\theta^2 + \psi^2 + \phi^2}$.

2.3 Constitutive relation of facets

Recalling the kinematics of 2.2, any point in the current configuration is

$$\mathbf{x^t}(\xi, \eta, \zeta) = \mathbf{x_m^t}(\xi, \eta) + \zeta\, \mathbf{n^t}(\xi, \eta).$$

With $(\cdot)_{,\xi}$ and $(\cdot)_{,\eta}$ denoting partial derivatives with respect to the mid-surface parameters, the deformation gradient is assembled column-wise and expanded to the first order in ζ:

$$F \;\approx\; \underbrace{\begin{bmatrix} x^t_{,\xi} & x^t_{,\eta} & n^t \end{bmatrix}}_{F_m} + \zeta\, \underbrace{\begin{bmatrix} n^t_{,\xi} & n^t_{,\eta} & 0 \end{bmatrix}}_{F_n}. \tag{S8}$$

The Green–Lagrange strain is

$$E = \tfrac{1}{2}\big(F^T F - I\big) \;\approx\; E^0 + \zeta\, E^1, \tag{S9}$$

with

$$E^0 = \tfrac{1}{2}\big(F_m^T F_m - I\big), \qquad E^1 = \tfrac{1}{2}\big(F_m^T F_n + F_n^T F_m\big). \tag{S10}$$

Using the components of E^0 and E^1,

$$\boldsymbol{\varepsilon}_m = \begin{bmatrix} E^0_{11} \\ E^0_{22} \\ E^0_{12} + E^0_{21} \end{bmatrix}, \qquad \boldsymbol{\varepsilon}_b = \begin{bmatrix} E^1_{11} \\ E^1_{22} \\ E^1_{12} + E^1_{21} \end{bmatrix}, \qquad \boldsymbol{\varepsilon}_s = \begin{bmatrix} E^0_{13} + E^0_{31} \\ E^0_{23} + E^0_{32} \end{bmatrix}. \tag{S11}$$

Remark. $\boldsymbol{\varepsilon}_m$ is the membrane strain (mid-surface), $\boldsymbol{\varepsilon}_b$ the bending strain (first-order in ζ), and $\boldsymbol{\varepsilon}_s$ the transverse shear strain (Reissner–Mindlin).

For an isotropic plate/shell with thickness t, Young's modulus E, Poisson's ratio ν, and shear-correction factor k,

$$\begin{bmatrix} \boldsymbol{\sigma}_m \\ \boldsymbol{\sigma}_b \\ \boldsymbol{\sigma}_s \end{bmatrix} = \begin{bmatrix} D_m & & \\ & D_b & \\ & & D_s \end{bmatrix} \begin{bmatrix} \boldsymbol{\varepsilon}_m \\ \boldsymbol{\varepsilon}_b \\ \boldsymbol{\varepsilon}_s \end{bmatrix}, \tag{S12}$$

where

$$D_m = \frac{E\,t}{1-\nu^2} \begin{bmatrix} 1 & \nu & 0 \\ \nu & 1 & 0 \\ 0 & 0 & \frac{1-\nu}{2} \end{bmatrix}, \qquad D_b = \frac{t^2}{12} D_m, \qquad D_s = \frac{k\,E\,t}{2(1+\nu)} \begin{bmatrix} 1 & 0 \\ 0 & 1 \end{bmatrix}. \tag{S13}$$

2.4 Crease Modelling

We treat each crease Γ_c as an interface curve between two adjacent facets. We distinguish three interface types: a free hinge, a torsional spring with rest angle, and an angle-lock (welded) joint. Here, $(\cdot)^+$ and $(\cdot)^-$ denote fields evaluated on the two sides of Γ_c; $\mathbf{u}$ and $\boldsymbol{\varphi}$ are the nodal displacement and rotation parameters, respectively; $\theta(s)$ is the dihedral angle about the crease tangent $\mathbf{t}(s)$.

Free rotational joint (hinge).

$$\mathbf{u}^+(s) = \mathbf{u}^-(s) \quad \forall\, s \in \Gamma_c, \tag{S14}$$

that is, translational continuity is enforced along the crease, whereas the relative rotation $\boldsymbol{\varphi}^+(s) - \boldsymbol{\varphi}^-(s)$ is left free (no energetic penalty).

Torsional spring with rest angle.

$$W_c = \int_{\Gamma_c} \frac{1}{2} k_\theta(s) \left[\theta(s) - \theta_0(s)\right]^2 \mathrm{d}s, \tag{S15}$$

with distributed hinge moment $m_\theta(s) = k_\theta(s) [\theta(s) - \theta_0(s)]$. Translational continuity is still enforced: $\mathbf{u}^+(s) = \mathbf{u}^-(s)$.

Angle lock (welded joint).

$$\mathbf{u}^+(s) = \mathbf{u}^-(s), \qquad \boldsymbol{\varphi}^+(s) = \boldsymbol{\varphi}^-(s) \quad \forall\, s \in \Gamma_c, \tag{S16}$$

that is, both translational and rotational DOFs are made identical across the crease (equivalent to merging the two facet edge DOFs).

2.5 Assembly and Solving

With the facet and crease models specified, we assemble the global system and solve the nonlinear equilibrium. We solve the weak form of the governing equation for the adapted Reissner–Mindlin shell using the meshless discretisation introduced earlier:

$$\int_V \delta\boldsymbol{\epsilon} : \boldsymbol{\sigma} \, \mathrm{d}V = \delta W_{\text{ext}}, \tag{S17}$$

which leads to the discrete equilibrium

$$\mathbf{F}_{\text{int}} = \mathbf{F}_{\text{ext}}, \tag{S18}$$

with $\mathbf{F}_{\text{int}} = \int_V \mathbf{B}\, \boldsymbol{\sigma}\, \mathrm{d}V$ the internal force vector (including virtual forces due to constraints) and $\mathbf{F}_{\text{ext}}$ the external and virtual load contributions. The geometric matrix $\mathbf{B}$ is defined by $\mathbf{B} = \delta\boldsymbol{\epsilon}/\delta\mathbf{u}$.

Large rotations and efficiency. Origami facets often undergo significant rotations. The point coordinates on the 3D shell were updated using the full Rodrigues formula to obtain the exact positions and associated strain–stress evaluation. To reduce the cost, the variation $\delta\boldsymbol{\epsilon}$ entering $\mathbf{B}$ is expanded by a Taylor series, whereas the stress update still uses the exact rotation.

Block structure of the geometric matrix. We decomposed $\mathbf{B}$ into membrane, bending, and transverse shear parts:

$$\mathbf{B} = \begin{bmatrix} \mathbf{B}_m \\ \mathbf{B}_b \\ \mathbf{B}_s \end{bmatrix}, \qquad \mathbf{B}_m = \mathbf{A}_m \mathbf{G}_m, \quad \mathbf{B}_b = \mathbf{A}_b \mathbf{G}_b, \quad \mathbf{B}_s = \mathbf{A}_s \mathbf{G}_s. \tag{S19}$$

Degrees of freedom and crease compatibility. In the discretised Reissner–Mindlin formulation, each node has five active DOFs in the local frame (three translations and two rotations). Although a node initially has six global DOFs, the drilling rotation is not directly used after the local transformation, which can render the global system singular. A common remedy is to constrain the drilling DOF to zero. However, in origami, facets share nodes along creases, and the drilling rotation acquires a clear physical meaning (rigid rotation about the facet normal). Instead of suppressing it, we enforce compatibility and continuity along shared creases through the in-plane twist measure

$$\gamma = \frac{1}{2}\left(\frac{\partial u}{\partial \eta} - \frac{\partial v}{\partial \xi}\right). \tag{S20}$$

Therefore, the crease kinematics remain consistent while keeping the global system non-singular.

Numerical integration. We use Gaussian integration (GI) over background cells placed on each facet's parametric plane; GI points are distributed according to the chosen quadrature order (lower-left panel of Fig. S2). For improved stability and efficiency, we adopted stabilised conforming nodal integration (SCNI), wherein each RK node acts as an integration point. The facet boundary layout and interior RK nodes define a Voronoi cell that serves as the local integration domain (right panels of Fig. S2). To further smooth the bending strains, we replace the original shape-function gradient with a boundary integral via Green's theorem:

$$\Psi_{I,\alpha}(\mathbf{x}_I) = \frac{1}{\Omega_L} \int_{\Gamma_L} \Psi(\mathbf{x}_k)\, N_{\alpha k}\, \mathrm{d}\Gamma, \qquad \alpha \in \{\xi, \eta\}, \tag{S21}$$

where Ω_L and Γ_L are the area and boundary of the Voronoi cell, $\mathbf{x}_k$ are the smoothing points on Γ_L, and $N_{\alpha k}$ is the projection of the boundary normal onto direction α.

Newton–Raphson-based solution. Denote the global unknown vector by $\mathbf{u}$. The discrete equilibrium $\mathbf{F}_{\text{int}}(\mathbf{u}) = \mathbf{F}_{\text{ext}}$ can be solved via Newton–Raphson-based iteration.

At iteration k, we linearise $\mathbf{r}$ and solve for the increment $\Delta\mathbf{u}_k$ from

$$\mathbf{K}_t(\mathbf{u}_k)\,\Delta\mathbf{u}_k = -\mathbf{r}(\mathbf{u}_k), \qquad \mathbf{K}_t(\mathbf{u}) = \frac{\partial \mathbf{F}_{\text{int}}}{\partial \mathbf{u}} = \int_V \frac{\partial \mathbf{B}\,\sigma}{\partial \mathbf{u}}\,\mathrm{d}V, \tag{S22}$$

followed by the update $\mathbf{u}_{k+1} = \mathbf{u}_k + \alpha_k\,\Delta\mathbf{u}_k$ with an optional line search factor $\alpha_k \in (0, 1]$ to ensure the descent of $\|\mathbf{r}\|_2$ or the total potential. Convergence is declared when $\|\mathbf{r}(\mathbf{u}_{k+1})\|_2/\|\mathbf{F}_{\text{ext}}\|_2 \leq \varepsilon_r$ and/or $\|\Delta\mathbf{u}_k\|_2/\|\mathbf{u}_{k+1}\|_2 \leq \varepsilon_u$. Constraints (*e.g.*, crease compatibility/drilling measure γ) are enforced in the residual assembly so that the final state satisfies both equilibrium and kinematic continuity along the shared creases.

3 Geometry–Mechanics Compatibility Test

We assess geometry–mechanics compatibility by combining two requirements: the deformation must remain bending-dominated, and the recovered bending field must agree with the geometric target implied by the intended developable motion. Both quantities are evaluated from the converged shell solution without introducing any additional kinematic approximation.

Given the converged global displacement vector $\boldsymbol{u}$, the reference mid-surface data (geometry, thickness t, and material blocks), and the quadrature, all pointwise strains and stresses at the integration points follow from the standard operators of Section 2. In particular, using the strain–displacement blocks

$$\boldsymbol{\varepsilon}_m = \boldsymbol{B}_m\,\boldsymbol{u}, \qquad \boldsymbol{\varepsilon}_b = \boldsymbol{B}_b\,\boldsymbol{u}, \qquad \boldsymbol{\varepsilon}_s = \boldsymbol{B}_s\,\boldsymbol{u}, \tag{S23}$$

and the block-diagonal constitutive law

$$\boldsymbol{\sigma}_m = \boldsymbol{D}_m\,\boldsymbol{\varepsilon}_m, \qquad \boldsymbol{\sigma}_b = \boldsymbol{D}_b\,\boldsymbol{\varepsilon}_b, \qquad \boldsymbol{\sigma}_s = \boldsymbol{D}_s\,\boldsymbol{\varepsilon}_s. \tag{S24}$$

we recover all field variables at each integration point (ξ, η) on the mid-surface.

Near-isometry and bending dominance. Define the strain-energy densities at an integration point

$$W_m = \tfrac{1}{2}\,\boldsymbol{\varepsilon}_m^{\mathsf{T}}\boldsymbol{D}_m\boldsymbol{\varepsilon}_m, \qquad W_b = \tfrac{1}{2}\,\boldsymbol{\varepsilon}_b^{\mathsf{T}}\boldsymbol{D}_b\boldsymbol{\varepsilon}_b, \qquad W_s = \tfrac{1}{2}\,\boldsymbol{\varepsilon}_s^{\mathsf{T}}\boldsymbol{D}_s\boldsymbol{\varepsilon}_s, \tag{S25}$$

and the integrated energies over the mid-surface Ω,

$$E_\alpha = \int_\Omega W_\alpha \, \mathrm{d}A \quad (\alpha \in \{m, b, s\}), \qquad E_{\mathrm{tot}} = E_m + E_b + E_s. \tag{S26}$$

Measure "bending dominance" with the energy fractions

$$r_m = \frac{E_m}{E_{\mathrm{tot}}}, \qquad r_s = \frac{E_s}{E_{\mathrm{tot}}}, \qquad r_b = \frac{E_b}{E_{\mathrm{tot}}} = 1 - r_m - r_s. \tag{S27}$$

For motions that remain close to the intended geometric mechanism, membrane and transverse-shear contributions should stay small compared with bending. We therefore require

$$r_m \le \eta_m, \qquad r_s \le \eta_s, \tag{S28}$$

with small tolerances η_m (*e.g.* $1\% - 5\%$ for near-isometry) and η_s (thin shells: typically $\lesssim 0.5\% - 2\%$ depending on formulation and t). If this screen fails, the motion is not interpreted as a geometry-compatible near-isometric response.

Geometric bending target and mismatch measures. From the developable-surface construction (guide curve and rulings with fixed incidence angle), we assemble the geometric target bending strain at the same integration points,

$$\boldsymbol{\varepsilon}_b^{\mathrm{geo}}(\xi, \eta),$$

expressed in the same tangent basis as the mechanical bending strain. A consistent surface-normal orientation is enforced before the comparison. We then quantify the mismatch between $\boldsymbol{\varepsilon}_b$ and $\boldsymbol{\varepsilon}_b^{\mathrm{geo}}$ by two nondimensional measures.

Energy-weighted mismatch. The primary metric is the energy-norm error

$$e_b^{(E)} = \left(\frac{\int_\Omega \left(\boldsymbol{\varepsilon}_b - \boldsymbol{\varepsilon}_b^{\mathrm{geo}}\right)^{\mathsf{T}} \boldsymbol{D}_b \left(\boldsymbol{\varepsilon}_b - \boldsymbol{\varepsilon}_b^{\mathrm{geo}}\right) \mathrm{d}A}{\int_\Omega \left(\boldsymbol{\varepsilon}_b^{\mathrm{geo}}\right)^{\mathsf{T}} \boldsymbol{D}_b \, \boldsymbol{\varepsilon}_b^{\mathrm{geo}} \, \mathrm{d}A + \delta} \right)^{1/2}, \tag{S29}$$

where $\delta > 0$ is a small safeguard (*e.g.* 10^{-10} in energy units).

Geometric mismatch. For applications that prefer a purely geometric normalisation, we also report

$$e_b^{(L)} = \left(\frac{\int_\Omega \|\boldsymbol{\varepsilon}_b - \boldsymbol{\varepsilon}_b^{\text{geo}}\|^2 \, \mathrm{d}A}{\int_\Omega \|\boldsymbol{\varepsilon}_b^{\text{geo}}\|^2 \, \mathrm{d}A + \delta} \right)^{1/2}. \tag{S30}$$

The characteristic length L may be taken as the panel span, RMS chord, or another problem-specific reference scale.

Acceptance criterion. We classify a response as geometry–mechanics compatible when

$$r_m \le \eta_m, \quad r_s \le \eta_s \quad \text{and} \quad e_b^{(E)} \le tol \quad (\text{or } e_b^{(L)} \le tol),$$

with a bending-misfit tolerance tol set by the application. Default tolerances are summarized in Table S4. All comparisons are carried out in a consistent tangent frame; if a different post-processing frame is used, the bending vectors are rotated before the metrics are evaluated. In Reissner–Mindlin formulations, a small but nonzero r_s is expected and should remain below η_s. If desired, an additional membrane strain cap (for example max principal$(\boldsymbol{\varepsilon}_m) \le 10^{-3}$) may be imposed as a stricter near-isometry filter.

Energy implication of the normalized misfit. Using the notation above, we denote the energy-weighted mismatch by $F_{\text{misfit}} \equiv e_b^{(E)}$. Both bending-strain fields are measured relative to the same reference curvature,

$$\boldsymbol{\varepsilon}_b = \boldsymbol{\kappa} - \boldsymbol{\kappa}_0, \qquad \boldsymbol{\varepsilon}_b^{\text{geo}} = \boldsymbol{\kappa}^{\text{geo}} - \boldsymbol{\kappa}_0, \tag{S31}$$

so the compatibility comparison also applies to facets with nonzero initial curvature. Define

$$\Delta\boldsymbol{\varepsilon}_b = \boldsymbol{\varepsilon}_b^{\text{geo}} - \boldsymbol{\varepsilon}_b, \qquad \|\boldsymbol{a}\|_{\boldsymbol{D}_b}^2 = \int_\Omega \boldsymbol{a}^{\mathsf{T}} \boldsymbol{D}_b \boldsymbol{a} \, \mathrm{d}A. \tag{S32}$$

For the energy-weighted normalization, the misfit is therefore

$$F_{\text{misfit}} = \frac{\|\Delta\boldsymbol{\varepsilon}_b\|_{\boldsymbol{D}_b}}{\sqrt{\|\boldsymbol{\varepsilon}_b\|_{\boldsymbol{D}_b}^2 + \delta_b}}, \tag{S33}$$

where $\delta_b > 0$ is a small numerical safeguard. The bending energies associated with the two fields are

$$U_b = \frac{1}{2}\|\boldsymbol{\varepsilon}_b\|_{\boldsymbol{D}_b}^2, \qquad U_b^{\text{geo}} = \frac{1}{2}\|\boldsymbol{\varepsilon}_b^{\text{geo}}\|_{\boldsymbol{D}_b}^2. \tag{S34}$$

Their difference satisfies

$$\begin{aligned}\left|U_b^{\text{geo}} - U_b\right| &= \left|\langle \boldsymbol{\varepsilon}_b, \Delta\boldsymbol{\varepsilon}_b \rangle_{\boldsymbol{D}_b} + \frac{1}{2}\|\Delta\boldsymbol{\varepsilon}_b\|^2_{\boldsymbol{D}_b}\right| \\ &\le \|\boldsymbol{\varepsilon}_b\|_{\boldsymbol{D}_b}\|\Delta\boldsymbol{\varepsilon}_b\|_{\boldsymbol{D}_b} + \frac{1}{2}\|\Delta\boldsymbol{\varepsilon}_b\|^2_{\boldsymbol{D}_b},\end{aligned} \tag{S35}$$

where the inequality follows from the Cauchy–Schwarz inequality. Substituting Eq. (S33) gives

$$\frac{\left|U_b^{\text{geo}} - U_b\right|}{U_b + \delta_b/2} \le 2F_{\text{misfit}} \frac{\|\boldsymbol{\varepsilon}_b\|_{\boldsymbol{D}_b}}{\sqrt{\|\boldsymbol{\varepsilon}_b\|^2_{\boldsymbol{D}_b} + \delta_b}} + F^2_{\text{misfit}} \le 2F_{\text{misfit}} + F^2_{\text{misfit}}. \tag{S36}$$

Consequently, the compatibility condition $F_{\text{misfit}} \le \text{tol}$ implies

$$\frac{\left|U_b^{\text{geo}} - U_b\right|}{U_b + \delta_b/2} \le 2\,\text{tol} + \text{tol}^2. \tag{S37}$$

When $U_b \gg \delta_b/2$, the denominator reduces to U_b to leading order. This establishes a direct connection between the field-level compatibility metric and bending-energy accuracy. Because the compatibility test is applied only after the membrane- and shear-energy fractions satisfy the near-isometry screen above, bending is the dominant facet deformation mode in the admitted cases. Reaction forces and stability characteristics nevertheless depend on derivatives and local features of the energy landscape and are therefore more sensitive near instability. Their accuracy is examined separately through the energy–displacement and force–displacement comparisons, stable-state locations, plateau forces, and target-shape results reported in Section 7.

4 1-DOF Model (Mode II) – Derivation

This section records the reduced derivation chain used by Mode II in the main text: isometric kinematics → principal curvature → bending energy → force and stiffness. Intermediate algebra is omitted whenever it does not alter the resulting design formulas.

4.1 Curved-crease origami with developable surfaces

We use the curved-crease metamaterial in the main text as the representative developable case. Let the reference surface be

$$S_0(u,v) = \begin{bmatrix} u \\ f(u) \\ 0 \end{bmatrix} + v \begin{bmatrix} 0 \\ -\cos(\pi/4) \\ -\sin(\pi/4) \end{bmatrix}, \tag{S38}$$

where u parameterises the guide curve, v follows the ruling direction, and $f(u)$ sets the reference profile.

Single-layer unit. Mode II assumes a quasi-isometric folding motion: the first fundamental form is preserved while the ruling direction rotates with the driven angle θ. For the representative case $\theta_0 = \pi/4$, the deformed tangents can be written as

$$S_u = \begin{bmatrix} \sqrt{1 + f'(u)^2 \left(1 - \frac{1}{2}\sec^2\theta\right)} \\ \frac{\sqrt{2}}{2} f'(u)\sec\theta \\ 0 \end{bmatrix}, \qquad S_v = \begin{bmatrix} 0 \\ -\cos\theta \\ -\sin\theta \end{bmatrix}. \tag{S39}$$

The surface remains developable, so one principal curvature is zero, $k_2 = 0$, and the nonzero principal curvature reduces to

$$k_1(\theta,u) = \frac{A\tan\theta}{\sqrt{B + C\sec^2\theta}}, \tag{S40}$$

with

$$\begin{aligned} A &= \frac{\frac{\sqrt{2}}{2} f''(u)}{(1 + 0.5 f'(u)^2)^{3/2}}, \\ B &= 1 + f'(u)^2, \\ C &= -0.5 f'(u)^2. \end{aligned} \tag{S41}$$

For a general rest angle θ_0, the same derivation gives the more useful form

$$A = \frac{\cos\theta_0\, f''(u)}{\left(1 + f'(u)^2 \sin^2\theta_0\right)^{3/2}}, \qquad B = 1 + f'(u)^2, \qquad C = -\cos^2\theta_0\, f'(u)^2. \tag{S42}$$

The mechanical response follows by evaluating the curvature change along the guide curve:

$$U(\theta) = \frac{1}{2}\int_0^T \left(k_1(\theta,u) - k_1(\theta_0,u)\right)^2 du, \tag{S43}$$

$$F(\theta) = \partial_\theta U(\theta), \qquad K(\theta) = \partial_{\theta\theta} U(\theta). \tag{S44}$$

Hence the single-layer ROM is fully governed by the geometry through $f'(u)$ and $f''(u)$ and by the rest angle through θ_0.

Double-layer unit. For the two-layer unit, compatibility between the outer and inner layers is enforced through

$$\theta_1 = \arccos(\alpha \cos\theta_2), \qquad \alpha = \frac{\cos\theta_{10}}{\cos\theta_{20}}, \tag{S45}$$

and the compatible end-shortening is measured by

$$H(\theta_2) = a_1 \sin\theta_1 - a_2 \sin\theta_2. \tag{S46}$$

Each layer retains the single-layer curvature form

$$k_1^{(i)}(\theta_i, u) = \frac{A^{(i)} \tan\theta_i}{\sqrt{B^{(i)} + C^{(i)} \sec^2\theta_i}}, \tag{S47}$$

with layer-specific coefficients $A^{(i)}, B^{(i)}, C^{(i)}$. The total energy is the sum of the two layer energies evaluated along the compatible angle pair, and the unit force is obtained by differentiating that total energy with respect to H. In this form, the role of interlayer locking is explicit: the response is controlled by the geometric map $\theta_1(\theta_2)$ rather than by a free superposition of two independent layer curves.

4.2 Curved-crease origami with doubly curved surfaces

Setup. Let the initial mid-surface be

$$S_0(u, v) = \begin{bmatrix} u \\ v \\ f(u) + g(v) \end{bmatrix},$$

with $f, g \in C^2$ in their parameter domains.

1-DOF isometric kinematics. We use the same 1-DOF construction as in the main text and parameterise the motion by a positive scalar $t > 0$:

$$S_u^t(u) = \begin{bmatrix} \sqrt{1 + f'^2(u)\left(1 - t^2\right)} \\ 0 \\ t\, f'(u) \end{bmatrix}, \qquad S_v^t(v) = \begin{bmatrix} 0 \\ \sqrt{1 + g'^2(v)\left(1 - t^{-2}\right)} \\ g'(v)/t \end{bmatrix}. \tag{S48}$$

These tangents preserve the first fundamental form exactly:

$$\langle S_u^t, S_u^t \rangle = 1 + f'^2, \qquad \langle S_v^t, S_v^t \rangle = 1 + g'^2, \qquad \langle S_u^t, S_v^t \rangle = f'g'.$$

Surface reconstruction and curvature. Because S_u^t depends only on u and S_v^t only on v, the cross derivatives vanish and the deformed surface is path independent. A compatible surface $S^t(u, v)$ therefore exists up to rigid translation. The curvature is then computed from the second fundamental form associated with (S48); in particular, the mixed coefficient is zero, $M = 0$, so the principal curvatures follow directly from the corresponding L and N terms.

Energy, force and stiffness. Given the bending block $\mathbf{D}_b$ defined in Section 2 and a reference state t_0, define the bending strain vector $\boldsymbol{\kappa}(t)$ on the facet. The Mode II energy is

$$U(t) = \frac{1}{2} \iint_\Omega \big(\boldsymbol{\kappa}(t) - \boldsymbol{\kappa}(t_0)\big)^\top \mathbf{D}_b \big(\boldsymbol{\kappa}(t) - \boldsymbol{\kappa}(t_0)\big)\, dA, \quad dA = \|S_u^t \times S_v^t\|\, du\, dv. \tag{S49}$$

Differentiating along the driving coordinate gives

$$U'(t) = \iint_\Omega \big(\boldsymbol{\kappa}(t) - \boldsymbol{\kappa}(t_0)\big)^\top \mathbf{D}_b\, \partial_t \boldsymbol{\kappa}(t)\, dA, \tag{S50}$$

$$U''(t) = \iint_\Omega \Big[\big(\partial_t \boldsymbol{\kappa}\big)^\top \mathbf{D}_b \big(\partial_t \boldsymbol{\kappa}\big) + \big(\boldsymbol{\kappa} - \boldsymbol{\kappa}_0\big)^\top \mathbf{D}_b\, \partial_{tt} \boldsymbol{\kappa} \Big] dA. \tag{S51}$$

For a prescribed loading protocol, we compute the actuation–parameter map $\delta = \delta(t)$ and report

$$F(\delta) = \frac{dU}{d\delta} = \frac{U'(t)}{\delta'(t)}, \qquad K(\delta) = \frac{d^2U}{d\delta^2} = \frac{U''(t)\,[\delta'(t)] - U'(t)\,\delta''(t)}{[\delta'(t)]^3}.$$

Thus the doubly curved case uses the same logic as the developable case: once the admissible 1-DOF kinematic family is fixed, the mechanical response is obtained by evaluating the bending energy along that single coordinate.

4.3 Curved-crease origami with general ruling distributions

The explicit single-degree-of-freedom (1-DOF) expressions used in Section 4.1 are specializations of a common differential-geometric construction. Consider two developable panels joined along a curved crease $\mathbf{r}_0(u)$. On each side $a \in \{+, -\}$, the panel is represented as a ruled surface

$$\mathbf{r}^a(u, v) = \mathbf{r}_0(u) + v\mathbf{d}^a(u), \qquad \mathbf{d}^a = \frac{\eta^a \mathbf{e}_1^a + \mathbf{e}_2^a}{\sqrt{1 + (\eta^a)^2}}, \qquad \eta^a = \cot\alpha^a, \tag{S52}$$

where α^a is the angle between the crease tangent and the ruling, $\mathbf{e}_1^a = \mathbf{t}$ is the unit crease tangent, and $\mathbf{e}_2^a$ is tangent to the corresponding panel. The parameter u need not be arc length; we write $\dot{s} = \|\mathbf{r}_0'(u)\|$.

Let β^a denote the rotation from the Frenet frame of the crease to the Darboux frame of side a. The geodesic curvature, normal curvature, and geodesic torsion along the crease are

$$\kappa_g^a = \kappa \cos\beta^a, \qquad \kappa_n^a = -\kappa \sin\beta^a, \qquad \tau_g^a = \frac{(\beta^a)'}{\dot{s}} + \tau, \tag{S53}$$

where κ and τ are the curvature and torsion of $\mathbf{r}_0$. Imposing developability on each ruled panel gives

$$(\mathbf{r}_0' \times \mathbf{d}^a) \cdot (\mathbf{d}^a)' = 0 \quad \iff \quad \eta^a \kappa_n^a + \tau_g^a = 0 \quad \iff \quad (\beta^a)' = \dot{s}\big(\eta^a \kappa \sin\beta^a - \tau\big). \tag{S54}$$

Thus, the crease geometry and ruling field enter through a common first-order initial-value problem rather than through a pattern-specific kinematic ansatz.

For a Euclidean curved crease, the intrinsic geodesic curvatures on its two sides satisfy

$$\kappa_g^+ + \kappa_g^- = 0. \tag{S55}$$

Choosing the branch $\beta^- = \pi - \beta^+$ and eliminating τ between the two copies of Eq. (S54) yields

$$(\beta^+)' = \frac{\dot{s}}{2}\kappa_g^+ \tan\beta^+ \big(\eta^+ - \eta^-\big). \tag{S56}$$

Under isometric folding, $\kappa_g^+(u)$ and the prescribed ruling fields $\eta^\pm(u)$ are invariant. Consequently, one scalar boundary value

$$q \equiv \beta^+(u_0), \qquad \beta^+(u_0; q) = q, \tag{S57}$$

determines $\beta^+(u; q)$ along the entire crease through Eq. (S56). The remaining geometric quantities follow from $\beta^- = \pi - \beta^+$, $\kappa = \kappa_g^+/\cos\beta^+$, and $\tau = \eta^+\kappa\sin\beta^+ - (\beta^+)'/\dot{s}$; integration of the Frenet–Serret equations and Eq. (S52) then reconstructs both panels up to a rigid-body motion. This defines

the common geometric map

$$(\mathbf{p}, q) \longmapsto \mathbf{X}^{\text{geo}}(\mathbf{p}, q) \longmapsto \boldsymbol{\kappa}^{\text{geo}}(\mathbf{p}, q), \tag{S58}$$

where $\mathbf{p}$ collects the crease and ruling geometry, while q is the single driven coordinate. The driven fold angle θ used in the main text may equivalently be chosen as a monotonic reparameterization of q.

The corresponding reduced energy is evaluated along this geometric path,

$$U_{\text{red}}(q; \mathbf{p}) = \frac{1}{2} \int_{\Omega} \left(\boldsymbol{\kappa}^{\text{geo}}(q; \mathbf{p}) - \boldsymbol{\kappa}^{0}\right)^{\mathsf{T}} \mathbf{D}_{b} \left(\boldsymbol{\kappa}^{\text{geo}}(q; \mathbf{p}) - \boldsymbol{\kappa}^{0}\right) \mathrm{d}A, \tag{S59}$$

with $\boldsymbol{\kappa}^{0}$ denoting the curvature of the stress-free reference state. For multi-panel or multilayer assemblies, the component energies have the same form and are summed after geometric or architectural constraints relate their local coordinates. The explicit coupling expressions used in Section 5 are closed-form realizations of these constraints rather than changes in the underlying modeling principle.

Equations (S52)–(S59) generate a candidate 1-DOF family; they do not assume that a compliant structure follows this path mechanically. That admissibility is determined independently by comparing the resulting geometric bending field with the high-fidelity solution using the geometry–mechanics compatibility criterion of Section 3 and the tolerance selected for the design objective. Only when $F_{\text{misfit}} \leq \text{tol}$ is the reduced family admitted for subsequent design-space exploration.

The Euclidean matching condition in Eq. (S55) is used for the representative developable case considered in Section 4.1; other intrinsic crease prescriptions require their corresponding cross-crease matching relation. Likewise, the doubly curved example in Section 4.2 uses a different explicit surface map. These differences alter the analytical realization of $\mathbf{X}^{\text{geo}}$, but not the common sequence of geometric generation, curvature and energy evaluation, and compatibility qualification.

5 Inverse Design Method

We adopt a structure-aware inverse-design pipeline rather than treating the reduced model as a black-box evaluator. Mode II is used in two complementary ways: as a fast constrained forward model inside standard optimisation routines, and as an interpretable reduced model that exposes

low-dimensional relations between geometry, layered architecture, and target response. In practice, the second route provides the main design logic, while optimisation is used to refine feasible candidates.

In the main text, we use this strategy for both force–displacement design and target-shape fitting. Because the Mode II reduced-order model (ROM) is extremely inexpensive to evaluate, it can be embedded directly in constrained search. More importantly, its closed-form structure often reveals which parameter combinations control the target behaviour before any solver is launched.

Below, we first state the optimisation formulations used when a final numerical fit is needed, and then summarise the equation-driven constructions that were used to guide the main-text designs.

5.1 Optimization as a refinement step

Let $p \in \mathcal{P}$ denote the vector of design parameters, and let $u_d \in \mathcal{U}$ be the user-prescribed actuation (*e.g.*, a displacement input). The Mode II ROM provides the forward maps

$$(p, u_d) \longmapsto F(u_d; p) \in \mathbb{R}, \tag{S60}$$

and

$$(p, u_d) \longmapsto \mathbf{x}(u_d; p) \in \mathbb{R}^{n_x}, \tag{S61}$$

returning the reaction force and deformed configuration, respectively.

Two representative inverse problems are as follows:

Force–displacement matching. Given a target curve $F_\star(u)$ sampled at $\{u_k\}_{k=1}^N$ with positive weights $\{w_k\}$, find p that minimizes

$$\min_{p \in \mathcal{A}} \sum_{k=1}^{N} w_k \left(F(u_k; p) - F_\star(u_k)\right)^2, \tag{S62}$$

where $\mathcal{A} \subseteq \mathcal{P}$ is the admissible set induced by the geometric and mechanical constraints.

Shape matching at a prescribed actuation. Given a target configuration $\mathbf{x}_\star$ at a user-defined actuation u_{user}, solve

$$\min_{p \in \mathcal{A}} \left\|\mathbf{x}(u_{\text{user}}; p) - \mathbf{x}_\star\right\|_2^2. \tag{S63}$$

These objectives serve as a numerical fallback when a direct parameter-to-response construction is unavailable, and as a final refinement step once a promising architecture has already been identified. We use MATLAB's Optimization Toolbox, combining the global `ga` solver for coarse exploration with `fmincon` for local refinement. In all cases, the search is restricted to the admissible set $\mathcal{A}$ and, whenever possible, warm-started from the equation-driven constructions described next. In this way, optimisation improves an already interpretable design rather than replacing the design logic.

5.2 Equation-driven inverse design (non–black-box)

This is the main design route used in the manuscript. Instead of asking the optimiser to discover the response structure from scratch, we express the target behaviour through a small set of solvability conditions that link layer parameters, compatible motions, and aggregate response. These relations provide interpretable seeds and, in many cases, already explain why a target is feasible or infeasible.

Prescribed mechanical curve design. Building on the analysis in Section 8.2, the design of programmable force–displacement curves can be recast as controlling how the two coupled layers contribute to the total energy and force. Varying the layer thickness is the most direct way to tune these weights while leaving the rest of the geometry nearly unchanged. Let p_ℓ be the geometric/hinge parameters of layer $\ell \in \{1, 2\}$, let $t_\ell > 0$ denote its thickness, and let $F_{L\ell}(u; p_\ell)$ be the layer-ℓ reaction force under actuation u.

(i) Boundary-locked (parallel-like) coupling. When boundary kinematics are constrained so that both layers experience the same actuation u, the total response is well approximated by a weighted superposition

$$F_{\text{tot}}(u; p_1, p_2, t_1, t_2) \;=\; w_1(t_1)\, F_{L1}(u; p_1) \;+\; w_2(t_2)\, F_{L2}(u; p_2), \tag{S64}$$

where $w_\ell(t_\ell) > 0$ is an effective stiffness weight that monotonically increases with t_ℓ and is calibrated once from the Mode II ROM. Under the admissible-angle and monotonicity/convexity conditions summarized in Section 8.2, the locked coupling in Eqn. (S64) cannot generate multistability or multiple plateau responses on its own: linear combinations of admissible single-layer curves remain in the same qualitative class. This observation is useful at the design stage because it rules out entire target families before any numerical search is attempted.

(ii) Series coupling of multi-unit two-layer modules and deformation sequencing. To realize multi-plateau or multistable responses, the additional freedom is introduced at the architectural level: multiple two-layer units are assembled in series. Each unit remains boundary-locked internally and is described by its own Mode II ROM, but different units are not kinematically locked to one another. The common axial force and additive displacement then allow deformation sequencing across units.

Unit model. For unit $j \in \{1, \ldots, n\}$, let $u_j \geq 0$ be its displacement, $p^{(j)} = (p_1^{(j)}, p_2^{(j)})$ its layer parameters, and $t^{(j)} = (t_1^{(j)}, t_2^{(j)})$ the layer thicknesses. The Mode II-reduced model provides the unit-level response as a weighted superposition of its two layers:

$$F^{(j)}(u_j; p^{(j)}, t^{(j)}) = \sum_{\ell=1}^{2} w_\ell^{(j)}\big(t_\ell^{(j)}\big)\, F_{L\ell}^{(j)}\big(u_j; p_\ell^{(j)}\big), \tag{S65}$$

where $F_{L\ell}^{(j)}$ is the single-layer force–displacement curve of layer ℓ in unit j, and $w_\ell^{(j)}(t) > 0$ are effective stiffness weights calibrated once from the Mode II ROM.

Series laws across units. Let $u = \sum_{j=1}^{n} u_j$ be the global actuation, and let $F(u)$ denote the common axial force. Series coupling enforces

$$F(u) = F^{(1)}(u_1; p^{(1)}, t^{(1)}) = \cdots = F^{(n)}(u_n; p^{(n)}, t^{(n)}), \qquad \sum_{j=1}^{n} u_j = u. \tag{S66}$$

Equivalently, introducing allocation coefficients $\alpha_j(u) \in [0, 1]$ with $\sum_{j=1}^{n} \alpha_j(u) = 1$ and $u_j(u) = \alpha_j(u)\, u$, the equal-force condition becomes

$$F^{(1)}\big(\alpha_1(u)\, u\big) = \cdots = F^{(n)}\big(\alpha_n(u)\, u\big) = F(u). \tag{S67}$$

Target matching at anchors. Given a target curve $F_\star(u)$ on $[0, U]$, we enforce matching at anchors $\{u^{(m)}\}_{m=1}^{M}$ by solving the small systems

$$\begin{aligned} &F^{(1)}\big(u_1^{(m)}; p^{(1)}, t^{(1)}\big) = \cdots = F^{(n)}\big(u_n^{(m)}; p^{(n)}, t^{(n)}\big) = F_\star\big(u^{(m)}\big), \\ &\sum_{j=1}^{n} u_j^{(m)} = u^{(m)}, \qquad u_j^{(m)} \geq 0 \ \ (j = 1, \ldots, n). \end{aligned} \tag{S68}$$

The unknowns in (S68) are the per-anchor splits $\{u_j^{(m)}\}$ together with the design parameters $\{p^{(j)}, t^{(j)}\}$. Solving these coupled conditions makes clear which unit is responsible for each segment of the target response and provides a structured initial design before any further optimisation.

By tailoring the mechanical response of each unit and then combining the units in series, the prescribed target curve can be approximated with a design that remains interpretable at both the unit and architecture levels.

Inverse design of shape for curved-crease origami. For shape design, the same interpretability is used geometrically rather than mechanically. Standard periodic-origami fitting typically relies on repeating a unit cell and approximating the target surface through piecewise curvature along one or two principal directions. In contrast, developable curved-crease origami generates a continuous curvature along one principal direction, so a one-dimensional tessellation in the transverse direction is often sufficient to build a smooth approximation with far fewer cells.

Mode II makes the map from local curved-crease geometry to the folded surface explicit. We use that map to fit canonical target geometries, delimit the attainable design space, and construct non-periodic patterns for more irregular shapes. The value of the reduced model here is not only speed, but also the ability to identify which geometric edits move the folded surface in the desired direction.

Representative shape-design demonstrations (Fig. 5). For the periodic examples in Fig. 5a–c, the target doubly curved surfaces are prescribed through their representative sectional geometry and optimized at full folding, so that the resulting crease layouts can be compared directly with the folded target shapes. For the aperiodic examples in Fig. 5d,e, the target cross-sectional outline and the side pattern are specified separately and then combined into a flat crease layout. The letter-shaped demonstrations serve as representative proofs of concept for this periodic-to-aperiodic extension: they show that the cross-section and side pattern can be designed in a decoupled manner while preserving a geometrically compatible folded realization.

5.3 Representative computational-cost benchmark

Because wall-clock computational cost depends on discretisation resolution, solver implementation, load-step density, convergence tolerances, and hardware, the following comparison is intended as a controlled representative benchmark rather than a universal timing standard for all simulation-centric inverse-design methods. We consider the multistable response-design problem in Fig. 4f of the main text. The direct numerical workflow couples an Abaqus/Standard shell model using S4R elements with MATLAB's `fmincon`, whereas the reduced workflow couples the proposed 1-DOF model with the same optimiser. Both calculations use the same target response, initial design, parameter bounds, objective function, and convergence criteria.

The three-unit FE model was discretised using 2,304 S4R elements (768 per 2L unit), corresponding to 14,400 active degrees of freedom, as reported by the Abaqus message-file field *NUMBER OF EQUATIONS* = 14400. Each response curve was evaluated at 21 load points using the normalised loading sequence 0:0.05:1, corresponding to 20 nominal load increments. The reduced model contained one reduced degree of freedom. All calculations were performed using Abaqus 2020 and MATLAB R2022a on an Intel Core i9-10900K CPU at 3.70 GHz (10 cores/20 threads), with 32 GB nominal RAM (31.83 GiB system-visible). A single CPU core was used for the calculations, with the Abaqus jobs run using `cpus=1`. These degrees-of-freedom counts are reported only to indicate the source of the model-size reduction and are not assumed to determine computational time universally.

As shown in Fig. S9a, the two workflows reach essentially the same final loss, whereas the reduced-model-driven optimisation is completed in 12.03 s, compared with 653.473 s for the FE-driven optimisation, corresponding to an approximately 54.32-fold reduction in wall-clock time for this benchmark. Of the FE total, 652.136 s is associated with Abaqus energy evaluation and 1.337 s with the common `fmincon` non-energy overhead across the same 23 iteration points. The iteration-wise decomposition in Fig. S9b further shows that the optimiser-internal computation is small in both workflows and that the difference arises primarily from energy evaluation. The reduced-model evaluation remains below 0.6 s throughout the optimisation, whereas the FE evaluation varies substantially among design iterations and reaches 116.56 s in the most computationally demanding iteration. Thus, under the controlled settings considered here, the measured acceleration can be attributed primarily to replacing repeated nonlinear FE analyses with the compatible 1-DOF model

rather than to differences in the optimisation setup. Compatibility qualification is performed once before the reduced model is admitted for this parameterised design family.

5.4 Parametric extrapolation of the compatibility-qualified reduced model

The compatibility test is intended to determine the admissibility of a reduced description for a parameterised design family, rather than to assert universal validity from a single configuration. To examine how reliably a baseline qualification transfers within such a family, we first verified that the baseline curved-crease model passed the compatibility test and then parameterised its geometry by H and β, while retaining the same boundary conditions. We sampled $H \in [5, 12]$ and $\beta \in [-1, 1]$ on a 28×28 tensor-product grid, giving 784 geometries. For every geometry, the reduced 1-DOF prediction was compared with its corresponding high-fidelity result using the compatibility measure and energy comparison defined in Section 3.

Figure S10 maps the absolute relative energy difference over the sampled parameter domain. The red star denotes the baseline geometry, while the four coloured corner markers identify representative extrapolated geometries at the limits of the H–β domain. The surrounding panels show these four geometries and their corresponding deformed configurations under the same boundary conditions, illustrating the geometric variation covered by the extrapolation study.

Figure S11 relates F_{misfit} to the absolute relative energy difference for the same 784 geometries, with colour denoting H. All sampled cases remain within the adopted compatibility tolerance, $F_{\mathrm{misfit}} \leq \mathrm{tol} = 0.2$, and the energy difference does not exceed 2.5% throughout the grid. This accuracy is sufficient for the mechanical-metamaterial inverse-design tasks considered here, in which the reduced model is used for rapid candidate evaluation and the selected design can subsequently be checked by high-fidelity analysis. The results therefore support extrapolation of the baseline-qualified reduced model within the sampled H–β range and under unchanged boundary conditions; they do not imply unrestricted transfer to arbitrary geometries or loading conditions.

6 Experiments

6.1 Metamaterial fabrication, calibration, and testing (for Fig. 4)

All metamaterials in Fig. 4 were fabricated monolithically using direct 3D printing. Fold lines were realised as locally thinned "living hinges" of width w, so that the rotation concentrated in a narrow ligament of thickness t_c. Following a standard line-spring abstraction, each crease is modelled as a *rotation spring per unit crease length* with stiffness $\tilde{k}_\theta$ measured in $\mathrm{N\,rad^{-1}}$; *i.e.*, the bending moment density along the crease satisfies $M(s) = \tilde{k}_\theta\,(\theta - \theta_0)$.

As a physics-based *nominal* estimate, we adopt the plate-theory expression for a narrow flexure

$$\tilde{k}_\theta = \frac{E\,t_c^3}{12\,(1-\nu^2)\,w}. \tag{S69}$$

In practice, we tune $\tilde{k}_\theta$ by adjusting the local hinge thickness, t_c. Unless otherwise stated, the material parameters (TPU 95A) used in the reduced-order model were $E = 38\,\mathrm{MPa}$ and $\nu = 0.49$.

For each design in Fig. 4, we tested $n = 3$ independent specimens under identical boundary conditions and reported the envelope/mean response band.

Effective hinge stiffness used in Fig. 4. In the ROM, the crease line spring values used after calibration are summarised below (per unit crease length):

Geometric parameters for Fig. 4c (programmable stiffness). Each unit is a double-layer element with upper/lower ligament thicknesses (t_{L1}, t_{L2}) as listed; the other geometry and boundary conditions are identical across the three variants.

Layer-wise parameters for Fig. 4f (multistable metamaterial). Three stacked sublattices (from top to bottom), each with a double-layer unit characterised by upper/lower thickness (t_{L1}, t_{L2}) and rest fold angles $(\theta_{0,L1}, \theta_{0,L2})$.

Layer-wise parameters for Fig. 4h (multi-plateau metamaterial). *Notes.* (i) Eqn. (S69) is used as a physics-guided mapping from the local hinge thickness t_c to a target $\tilde{k}_\theta$; therefore, small, localised thickness edits are a controlled way to fine-tune the crease stiffness owing to cubic scaling. (ii) The effective values reported in Table S5 were used consistently in the ROM and were obtained

with a single calibration reused across Fig. 4. (iii) For each design, $n = 3$ specimens were tested under identical boundary conditions to obtain the experimental response band, as shown in Fig. 4.

6.2 Manipulator fabrication and module assembly (for Fig. 6b–f)

The origami manipulators in Fig. 6 were built through the unified "print–fold–heat–assemble" workflow shown in Fig. 6b. The flat origami patterns were printed on a Bambu Lab H2D using the manufacturer's PETG Basic and TPU 95A HF filaments. After folding into the prescribed three-dimensional geometry, the parts were thermally set with the aid of more heat-resistant ABS fixtures together with metal hardware such as screws and nuts to hold the encoded shape. The assembly was then placed in an oven at 55 °C for 8 h and subsequently cooled to room temperature before demolding and final assembly. This workflow was used for both polyline-based and curved-crease-based segment realizations so that different kinematic modules could be compared within the same overall fabrication route.

The deployed manipulator was assembled from two geometry-encoded components: a kinematic-path module and a task-specific end-effector. The path module was composed from the primitive motions in Fig. 6c, namely extension, bending, and twisting, whereas the end-effector was selected according to the intended grasping task. All demonstrations in Fig. 6f–i used the same cable-driven 1-DOF actuation architecture, allowing different path encodings and end-effectors to be recombined without redesigning the entire system.

6.3 Representative deployment and task demonstrations (for Fig. 6d–i)

For the deployment comparison in Fig. 6d,e, polyline and curved-crease segments with matched kinematic functions were actuated under the same loading scenario so that the effect of crease geometry on the deployment dynamics could be observed directly. The purpose of this comparison was to show how the same path-level function can be retained while the release behavior is tuned through the segment realization.

For the manipulator demonstrations in Fig. 6f–i, representative path encodings were selected from the broader programmable path space and paired with task-matched end-effectors. The demonstrations include open-space grasping and grasping in a pipe-like confined environment,

as shown in Fig. 6g,h and Movie S6. These tests are intended as representative system-level demonstrations of the design pipeline rather than a statistical benchmarking campaign.

6.4 Test setup

The quasi-static setup used for the metamaterial experiments in Fig. 4 is shown in Fig. S8.

7 Detailed Results of Mode I

7.1 Benchmark organization and boundary conditions

Figure S3 presents six representative Mode I benchmarks, whose boundary conditions are summarized in Table S2. Their validation results are discussed together with the additional Miura, Resch, and square-twist benchmarks in Section 7.3.

7.2 Details of Fig. 3 in the main text

The boundary conditions for the four cases in Fig. 3 are summarized in Table S3.

For Case IV (Fig. 3d), BC1 and BC2 represent two independent displacement-controlled loading modes. Under BC1, $U_z = 0$ is imposed on the lower boundary and $U_z = -u$ on the upper boundary, producing compression along the z-direction. Under BC2, $U_x = 0$ is imposed on one side boundary and $U_x = +u$ on the opposite side boundary, producing stretching along the x-direction.

7.3 Representative and Additional Benchmark Results

Kresling tube under compression and torsion. To validate the reliability of the high-fidelity reference analysis, we compared strain-energy–displacement (or rotation) responses with those from shell-based FEA. For the Kresling tube in Figure S3(a,b), four loading conditions were examined: axial compression, axial compression with fixed twist, prescribed twist with free axial motion, and prescribed twist with fixed axial displacement. Across all four conditions, Mode I captures the strain-energy evolution and the corresponding deformation sequences along the prescribed displacement or rotation path.

Cylinder waterbomb and large-scale waterbomb ring. The cylinder waterbomb in Figure S3(c) provides a geometrically more demanding benchmark under radial loading. Shell FEA loses convergence at large deformation because of severe mesh distortion, whereas Mode I remains stable and yields a smooth strain-energy path. The default bar-and-hinge model, used without case-specific calibration of equivalent hinge parameters, shows larger deviations and therefore highlights the parameter sensitivity of simplified modelling approaches. To assess scalability,

Figure S3(d) considers a 20-unit waterbomb ring relevant to variable-diameter wheels; Mode I handles the larger assembly without remeshing and captures its strain-energy evolution.

Curved-crease ribbon and tubes. The curved-crease examples in Figure S3(e,f) examine applicability beyond straight-crease benchmarks. For the ribbon under compression, Mode I matches the strain-energy trend from FEA while resolving the deformation sequence (i–iii) without planar-facet approximations. For the curved-crease tubes under axial compression, Mode I captures the mechanical responses across the different geometric configurations and provides geometry-exact curvature fields.

Miura pattern. The supplementary Miura examples add two pieces of information that are only summarised in the main text: stacked compression under two orthogonal loading directions and sheet bending about two orthogonal axes. Figure S4 documents the comparison among Mode I, shell FEA, and experiments for $2 \times 2 \times 2$ and $4 \times 4 \times 4$ stacks, so that the effect of loading direction and system size can be read together with the panel-wise boundary conditions. Figure S5 provides the corresponding bending responses and representative deformed shapes for a single-layer 6×6 sheet.

Resch pattern. The Resch examples are included to show the same comparison on a pattern with stronger facet bending. Figure S6(a,b) records the curved-sheet case, where shell FEA loses convergence at larger deformation while Mode I continues to provide a continuous response. Figure S6(c–e) then adds the stacked cases under axial compression and shear, together with the associated deformation and strain maps. The bar-and-hinge surrogate is retained as a qualitative reference for where facet bending becomes important.

Square-twist pattern. The square-twist example adds a tensile benchmark on a metasheet built from Type-3 square-twist units. Figure S7 collects the crease assignment, the loading setup, and the response/deformation maps under the prescribed tensile boundary conditions shown in panel (b). Its main role here is to document the exact benchmark setup used for the Mode I versus shell-FEA comparison on a tensile, metasheet-scale case.

8 Interpretability of Mode II

This section keeps only the consequences of the Mode II formulas that are directly used later in the design discussion. The goal is not to build a standalone theory of the reduced model, but to clarify which behaviours are permitted or excluded once the closed-form relations in Section 4 are fixed.

8.1 Single-layer implications for design

For a single layer, the principal curvature takes the form

$$k_1(\theta,u) = \frac{A\,\tan\theta}{\sqrt{B + C\,\sec^2\theta}}, \qquad A = \frac{\cos\theta_0\, f''(u)}{\left(1 + f'(u)^2 \sin^2\theta_0\right)^{3/2}}, \quad B = 1{+}f'(u)^2, \quad C = -\cos^2\theta_0\, f'(u)^2.$$

Admissible range and monotonicity. The denominator must remain positive, which gives the admissible interval

$$\cos^2\theta > \frac{\cos^2\theta_0}{1 + f'(u)^2} \quad\Longleftrightarrow\quad |\theta| < \arccos\!\left(\frac{|\cos\theta_0|}{\sqrt{1 + f'(u)^2}}\right). \tag{S70}$$

Within this interval,

$$\operatorname{sign}\!\left(\frac{\partial k_1}{\partial\theta}\right) = \operatorname{sign}(A) = \operatorname{sign}\!\left(\cos\theta_0\, f''(u)\right). \tag{S71}$$

Hence, once $f(u)$ and θ_0 are fixed, the curvature varies monotonically with the driving angle at fixed u. This is why a single compatible layer is effective for smooth stiffness programming but cannot realise arbitrary force profiles on its own.

Local stiffness and single-well tendency. Defining

$$U(\theta) = \frac{1}{2}\int_0^T \left(k_1(\theta,u) - k_1(\theta_0,u)\right)^2 \mathrm{d}u,$$

the tangent stiffness at the reference state satisfies

$$K(\theta_0) = \int_0^T \left(\frac{\partial k_1}{\partial\theta}(\theta_0,u)\right)^2 \mathrm{d}u > 0. \tag{S72}$$

Thus the rest state is locally stable. A stronger sufficient condition for global convexity is

$$\left|\left(k_1 - k_{10}\right)\frac{\partial^2 k_1}{\partial\theta^2}\right| \le \left(\frac{\partial k_1}{\partial\theta}\right)^2 \quad \text{for all admissible } \theta, u, \tag{S73}$$

which keeps the energy single-well over the admissible interval. These two relations explain why richer behaviours such as multistability or multiple plateau responses require an additional architectural ingredient beyond a single admissible layer.

8.2 Double-layer implications for architecture design

For a bilayer unit, the geometric compatibility map remains

$$\theta_1(\theta_2) = \arccos\bigl(\alpha \cos\theta_2\bigr), \qquad \alpha = \frac{\cos\theta_{10}}{\cos\theta_{20}} \in (0, 1]. \tag{S74}$$

The total energy can therefore be written in the driven coordinate of the inner layer as

$$U_{\mathrm{tot}}(\theta_2) = U_1\bigl(\theta_1(\theta_2)\bigr) + U_2(\theta_2). \tag{S75}$$

The interpretive point is that the outer layer contributes through the even round-trip map $\theta_1(\theta_2)$, whereas the inner layer can bias the energy through its own reference angle and stiffness scale. Their competition shifts the well position and changes the stiffness landscape. This is the mechanism behind the positive-, near-zero-, and negative-stiffness responses in the main text, and it also explains why relaxing geometric locking between units enables the multistable and multi-plateau architectures discussed in Section 5.2.

Figures S1 to S11

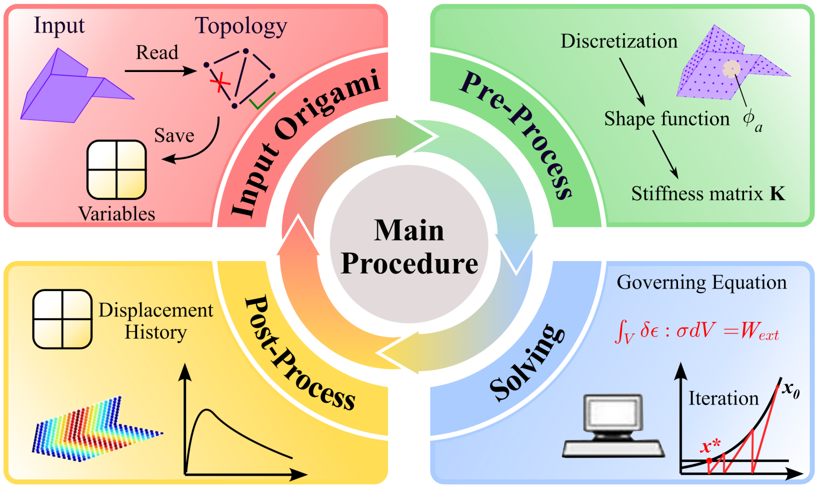

Figure S1. Main procedure of Mode I. Input origami, pre-process, solving, and post-process.

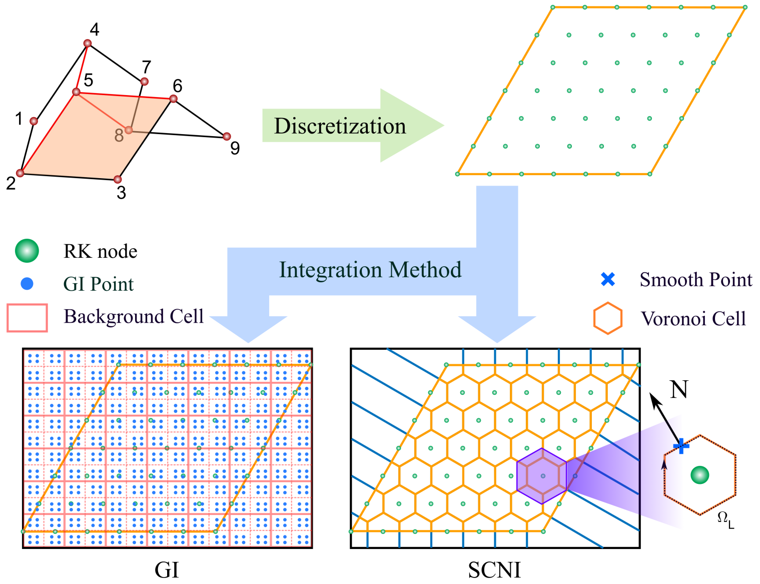

Figure S2. Integration strategy. Left: Gaussian integration over the background cells (GI). Right: SCNI with Voronoi cells built around RK nodes; smoothing points (filled) and outward normals are used in Eqn. (S21).

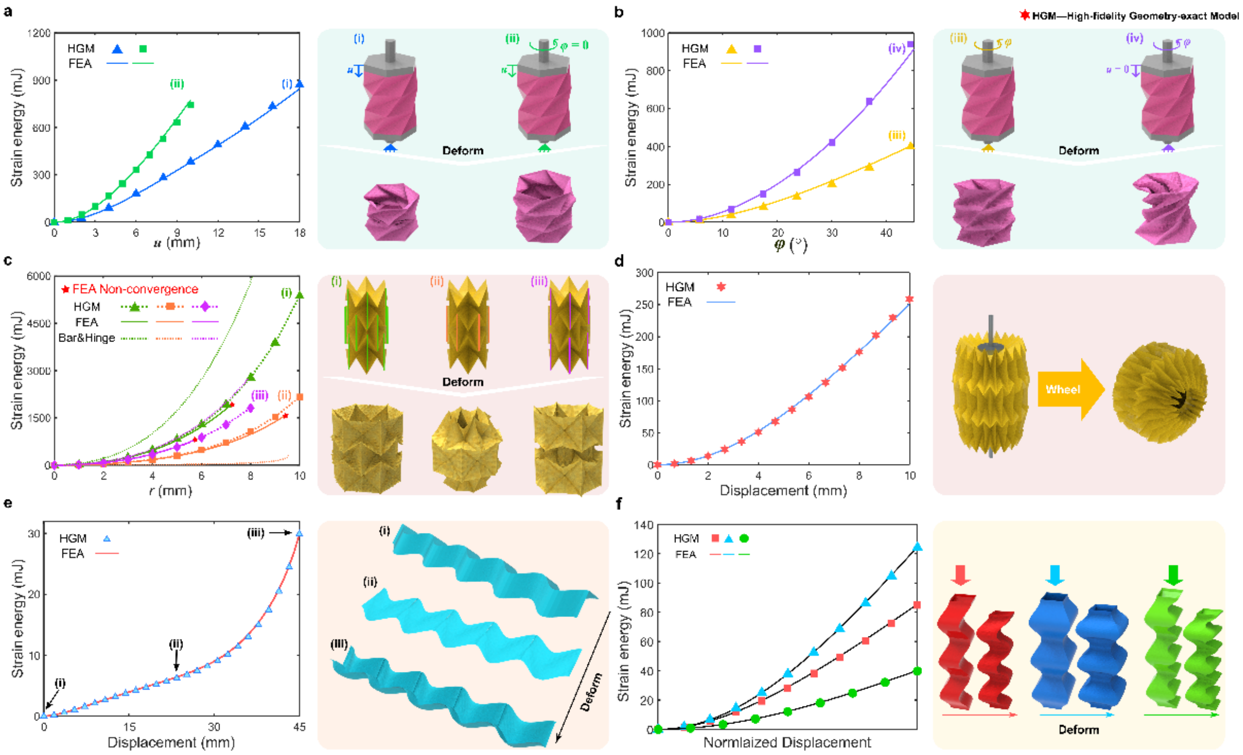

Figure S3. Geometry-exact analysis and validation of representative origami configurations. (a,b) Kresling origami tube under four loading modes: compression, compression with constrained twist, twist, and twist with constrained axial motion. The high-fidelity geometry-exact analysis (Mode I) reproduces the strain-energy trends and deformation sequences (i–iv). (c) Cylinder waterbomb under radial loading. Shell FEA fails to converge because of element distortion, whereas Mode I advances stably; the default bar-and-hinge model shows larger deviations. (d) Large-scale waterbomb ring comprising 20 units and relevant to variable-diameter wheels. (e) Curved-crease ribbon under compression, with deformed configurations (i–iii). (f) Axial compression of curved-crease tubes with different geometric configurations.

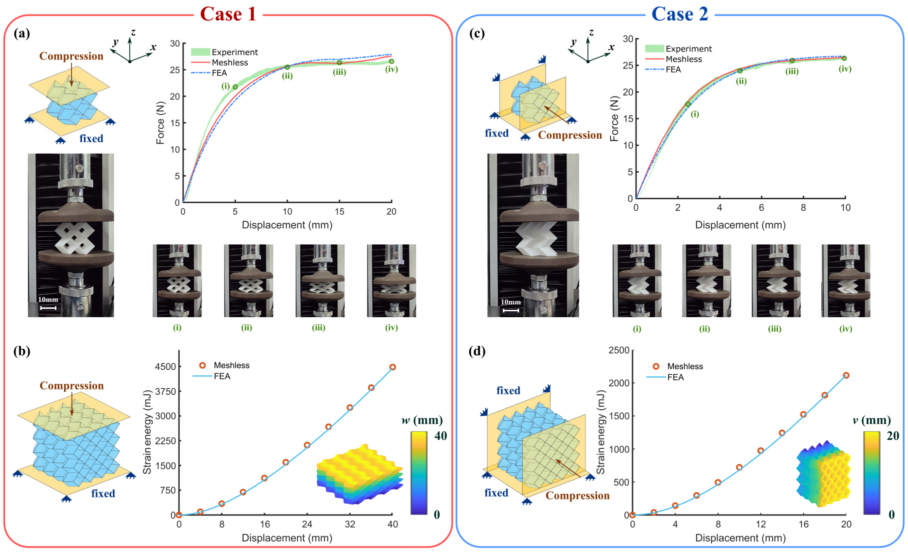

Figure S4. Miura stacked metamaterials under compression along two directions. (a,c) *2×2×2* stacks compressed along the z-direction (Case 1) and the y-direction (Case 2), respectively. Geometry-exact meshless analysis (Mode I) and shell FEA predict force–displacement curves that agree closely with quasi-static experiments on 3D-printed specimens; the light-green bands indicate the envelope from $n = 3$ nominally identical tests. (b,d) *4×4×4* stacks under the same boundary conditions as in Cases 1 and 2. The meshless method and FEA remain in close agreement and capture uniform layer-wise deformation across the lattice.

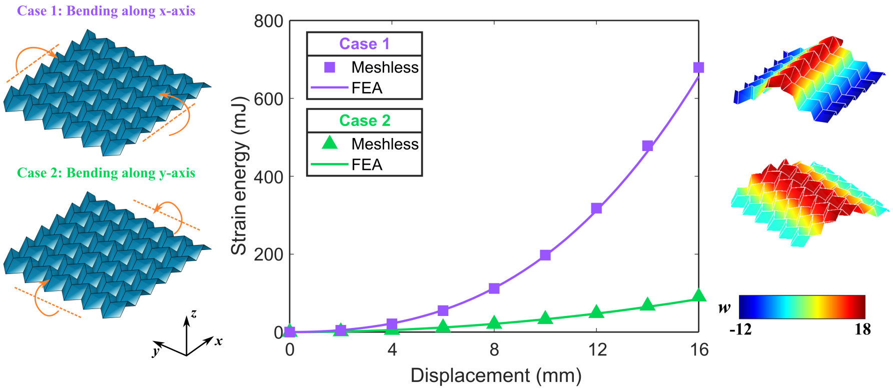

Figure S5. Bending of a 6×6 **Miura sheet about orthogonal axes.** Case 1: bending about the x-axis. Case 2: bending about the y-axis. For each case, strain–energy–displacement curves from geometry-exact meshless analysis (Mode I) and shell FEA are reported under identical boundary conditions, together with representative deformed shapes along the loading path.

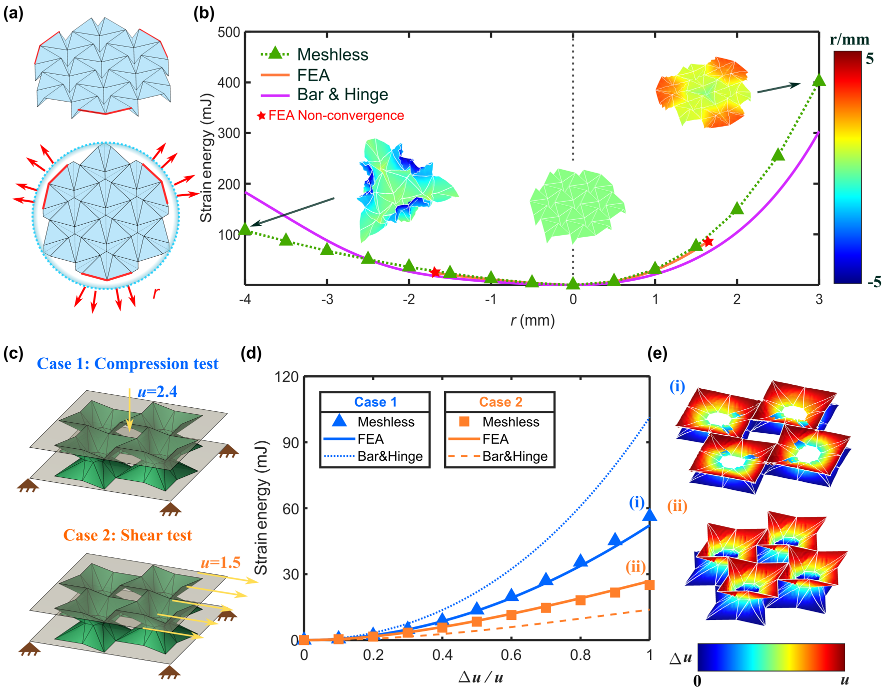

Figure S6. Resch pattern: curved sheet and stacked metamaterials. (a) Curved Resch unit with three radial tensile or compressive loading directions. (b) Strain–energy–displacement comparison for the curved sheet. (c) Stacked Resch metamaterials under axial compression (Case 1) and shear (Case 2). (d) Corresponding strain–energy responses from meshless analysis, shell FEA, and a bar-and-hinge surrogate. (e) Deformation and strain maps along the loading path.

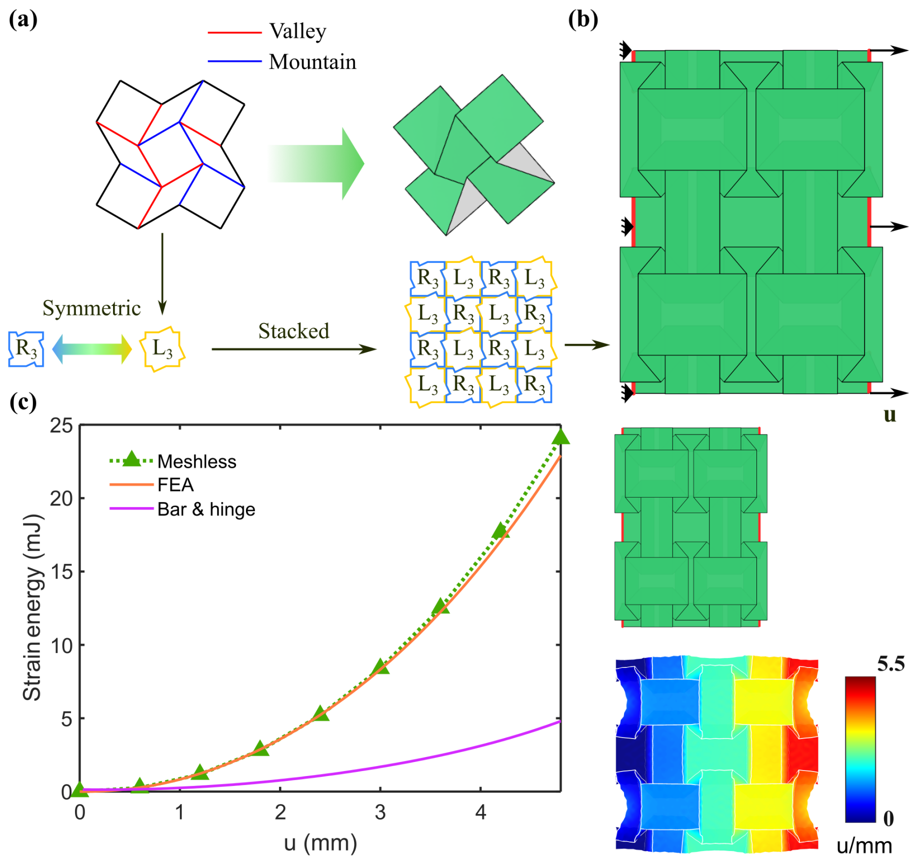

Figure S7. Square-twist meta-sheet under uniaxial tension. (a) Type-3 square-twist crease assignment and the 4×4 tessellation strategy using mirror-symmetric units (L_3 and R_3). (b) Boundary conditions for the tensile test. (c) Energy–displacement curves from geometry-exact meshless analysis (Mode I), shell FEA, and an uncalibrated bar-and-hinge model, together with representative displacement maps of the deformed shapes.

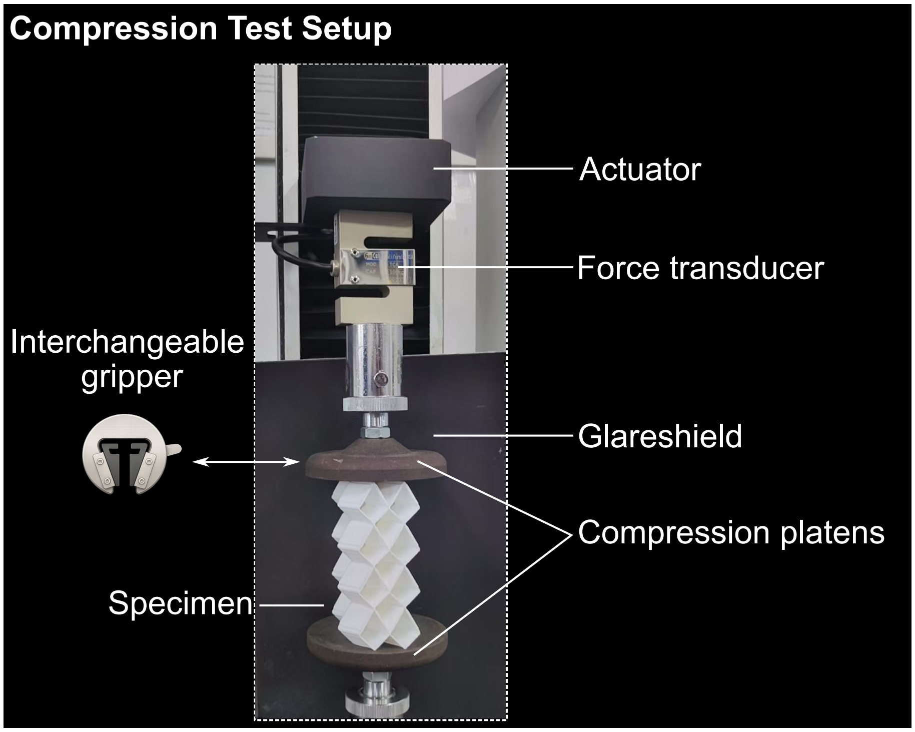

Figure S8. Universal testing machine used for the quasi-static experiments. Schematic of the loading and fixture setup.

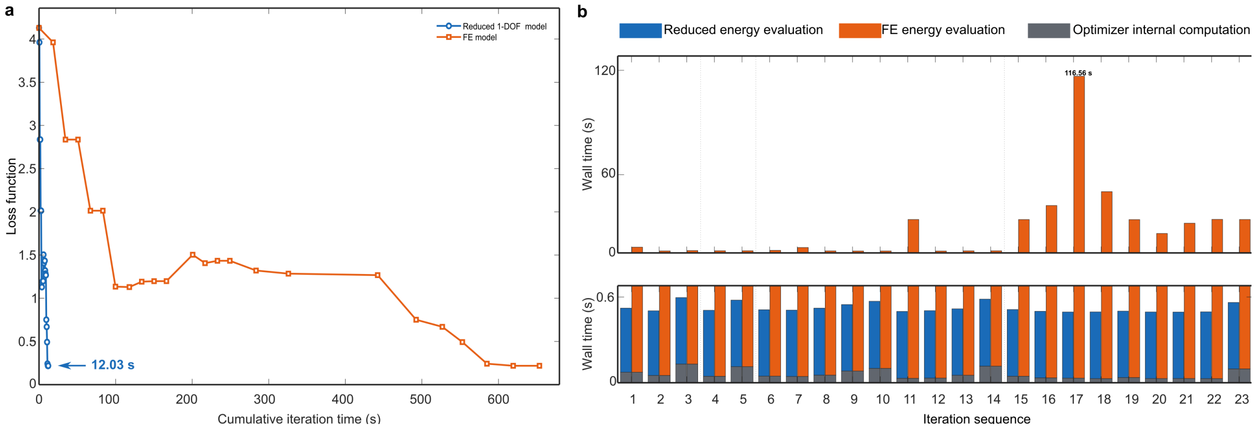

Figure S9. Representative computational-cost benchmark for the multistable response-design problem in Fig. 4f of the main text. (a) Loss function versus cumulative iteration time for optimisation driven by the reduced 1-DOF model and by the FE model. (b) Iteration-wise decomposition of the wall-clock time into reduced- or FE-model energy evaluation and optimiser-internal computation.

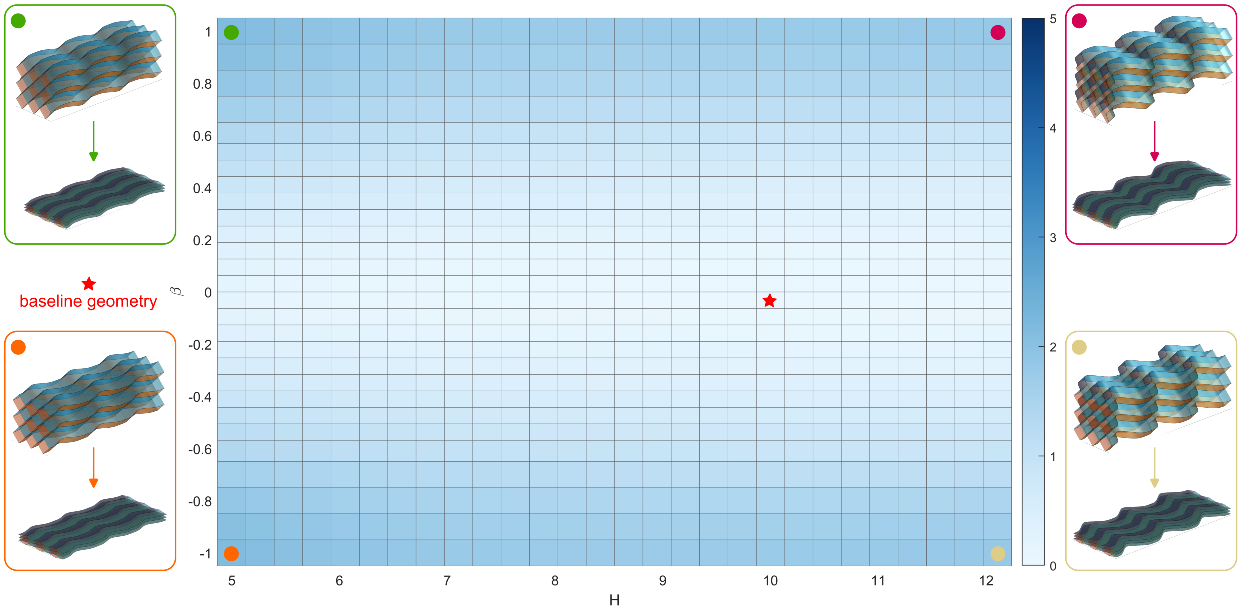

Figure S10. Parametric extrapolation of the compatibility-qualified reduced model. The central map shows the absolute relative energy difference over a 28×28 grid spanning $H \in [5, 12]$ and $\beta \in [-1, 1]$. The red star denotes the baseline geometry. The four coloured corner markers denote representative extrapolated geometries at the limits of the parameter domain; the surrounding panels show their geometries and corresponding deformed configurations under the same boundary conditions.

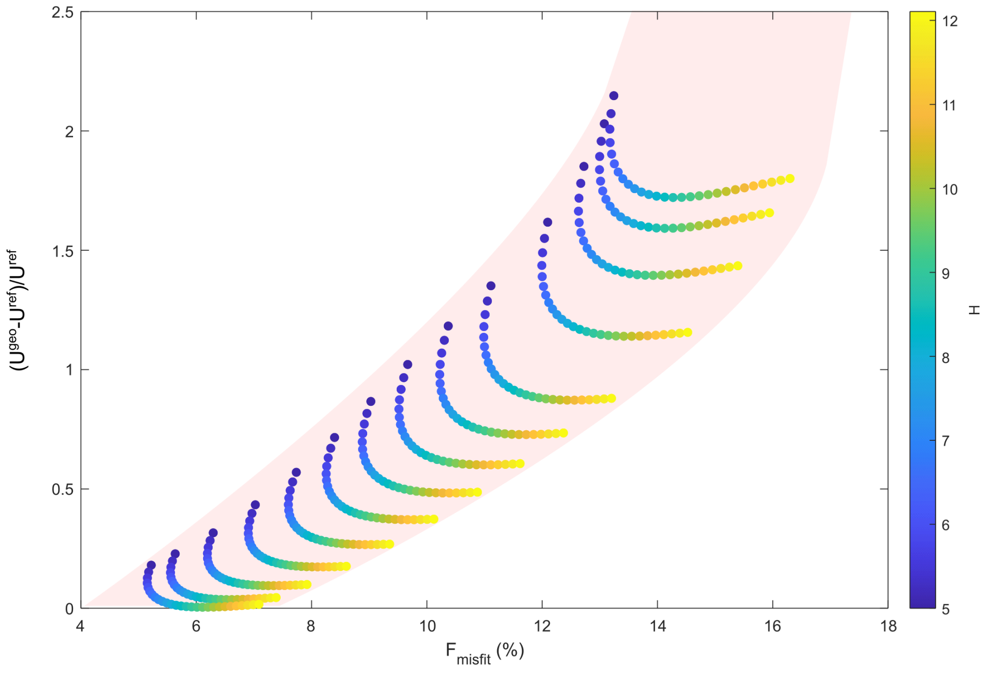

Figure S11. Relationship between normalised misfit and energy difference across the extrapolated family. Each point represents one of the 784 geometries in the H–β grid, with colour denoting H. All cases satisfy $F_{\text{misfit}} \leq \text{tol} = 0.2$, and the absolute relative energy difference remains below 2.5%.

Tables S1 to S8

Table S1. Notation and conventions used throughout the Supplementary Materials.

Symbol	Definition
Geometry & topology	
$\mathcal{V}$, $\mathcal{E}$, $\mathcal{F}$	Vertex set, crease-edge set, and face set of the planar graph
$S(u, v)$	Parametric surface; (u, v) are the surface parameters
Γ_c; s	Crease curve on S; arc-length along Γ_c
(ξ, η); ζ	Mid-surface parameters on a facet; signed through-thickness coordinate
$\mathbf{x}^0$, $\mathbf{x}^t$; $\mathbf{x}_m^0$, $\mathbf{x}_m^t$	Position of a material point; mid-surface position in the initial and deformed configurations
$\mathbf{n}^0$, $\mathbf{n}^t$	Director in the initial and deformed configurations
$\mathbf{t}$, $\mathbf{n}$, $\mathbf{b}$	Tangent, normal, and binormal along Γ_c
Rotations (Rodrigues)	
$\mathbf{R}^t$	Rotation tensor; $\mathbf{n}^t = \mathbf{R}^t \mathbf{n}^0$
$\mathbf{S}$; ω	Skew-symmetric matrix in the Rodrigues formula and the rotation magnitude
ϕ, θ, ψ	Components of the incremental rotation vector used to build $\mathbf{S}$
Discretization (RKPM)	
$\mathbf{x}_I$; N_P	Node I position on a facet and the number of nodes in the local patch
Ψ_I	Shape function associated with node I
$\mathbf{u}_I$; $\mathbf{u}^h$	Nodal displacement and interpolated displacement field
$H(\cdot)$	Polynomial basis column
$\phi_a(\cdot)$	Radial weight kernel with compact support a
$M(\mathbf{x})$; $c(\mathbf{x})$	RKPM moment matrix and coefficient vector $M^{-1}(\mathbf{x})H(\mathbf{0})$

Assembly

$\boldsymbol{\varepsilon},\ \boldsymbol{\sigma}$	Strain and stress
$\mathbf{F}_{\text{int}},\ \mathbf{F}_{\text{ext}}$	Internal and external force vectors
$\mathbf{B}$	Geometric matrix, $\mathbf{B} = \delta\boldsymbol{\epsilon}/\delta\mathbf{u}$
$\mathbf{B}_m, \mathbf{B}_b, \mathbf{B}_s$	Membrane, bending, and shear parts of $\mathbf{B}$
$\mathbf{A}_m\mathbf{G}_m,\ \mathbf{A}_b\mathbf{G}_b,\ \mathbf{A}_s\mathbf{G}_s$	Factorization of the three $\mathbf{B}$ blocks

Crease compatibility

γ	In-plane twist or drilling measure on a facet
$\theta(s),\ \theta_0(s)$	Dihedral angle and rest angle along Γ_c
$k_\theta(s)$	Torsional stiffness along the crease

Integration (GI/SCNI)

$\Omega_L,\ \Gamma_L$	Area and boundary of the Voronoi cell for an RK node
$\Psi_{I,\alpha}(\mathbf{x}_I)$	Smoothed gradient at the node via Green's formula
$N_{\alpha k}$	Projection of the boundary normal onto direction $\alpha \in \{\xi, \eta\}$

Nonlinear solve (Newton)

$\mathbf{r}(\mathbf{u})$	Residual vector: $\mathbf{r} = \mathbf{F}_{\text{int}} - \mathbf{F}_{\text{ext}}$
$\mathbf{K}_t$	Consistent tangent: $\mathbf{K}_t = \partial\mathbf{F}_{\text{int}}/\partial\mathbf{u}$
$\Delta\mathbf{u},\ \alpha$	Newton increment and optional line-search factor
$\varepsilon_r,\ \varepsilon_u$	Residual and displacement convergence tolerances

Geometry–mechanics compatibility test

Ω	Mid-surface domain used for area integrals in 3
$D_m,\ D_b,\ D_s$	Constitutive sub-blocks used in the shell law and in the compatibility test
$\boldsymbol{\varepsilon}_m,\ \boldsymbol{\varepsilon}_b,\ \boldsymbol{\varepsilon}_s$	Membrane, bending, and transverse-shear strain vectors
$\boldsymbol{\sigma}_m,\ \boldsymbol{\sigma}_b,\ \boldsymbol{\sigma}_s$	Stress or stress-resultant vectors for the three blocks

$W_m,\ W_b,\ W_s$	Strain-energy densities of the membrane, bending, and shear parts
$E_m,\ E_b,\ E_s$	Energies integrated over Ω for membrane, bending, and shear
E_{tot}	Total energy: $E_m + E_b + E_s$
$r_m,\ r_s,\ r_b$	Energy fractions: $r_\alpha = E_\alpha / E_{\mathrm{tot}}$ for $\alpha \in \{m, s, b\}$
$\eta_m,\ \eta_s$	Pre-screen tolerances for r_m and r_s
$\boldsymbol{\varepsilon}_b^{\mathrm{geo}}(\xi, \eta)$	Geometric target bending strain vector at (ξ, η)
$e_b^{(E)},\ e_b^{(L)}$	Dimensionless bending misfit measures
δ	Small positive safeguard constant used in the denominators
tol	Acceptance tolerance for the bending misfit
Reduced-order model (Mode II)	
$S_0(u, v)$	Reference mid-surface used in the 1-DOF constructions
$f(u),\ g(v)$	Geometry functions defining the guide/profile curves of the folded surface
$\theta,\ \theta_0$	Driven folding angle and rest angle of a compatible layer
$k_1,\ k_2$	Principal curvatures; for developable cases $k_2 = 0$
$A,\ B,\ C$	Geometry-dependent coefficients in the reduced curvature relation
$U,\ F,\ K$	Energy, reaction force, and tangent stiffness along the reduced path
$\theta_1,\ \theta_2;\ \alpha$	Compatible bilayer angles and their geometric ratio
$H(\theta_2)$	Compatible end-shortening of the bilayer unit
$\boldsymbol{\kappa}(t);\ t$	Bending-strain vector and 1-DOF path parameter in the doubly curved case
Inverse design	
$p,\ \mathcal{P},\ \mathcal{A}$	Design vector, parameter space, and admissible design set
$u_d,\ u_j$	Prescribed actuation and unit-level actuation in a multi-unit architecture
$F_\star(u),\ \mathbf{x}_\star$	Target force–displacement curve and target configuration
w_k	Sample weights used in force–displacement matching

Table S2. Boundary conditions for the six benchmark cases in Fig. S3. Global axis $\hat{z}$ is the axial direction.

Panel	Specimen	Attachments	Lower boundary	Upper boundary
a	Kresling tube	The top and bottom faces were kinematically tied to the rigid platens.	Bottom platen fixed: $U_{x,y,z} = 0,\ R_{x,y,z} = 0.$	Displacement-controlled $U_z = -u$; $U_{x,y} = 0,\ R_{x,y} = 0.$ (i) R_z free; (ii) $R_z = 0.$
b	Kresling tube (torsion)	Top and bottom faces kinematically tied to rigid platens.	Bottom platen fixed: $U_{x,y,z} = 0,\ R_{x,y,z} = 0.$	Torsion-driven $R_z = \varphi$ about $\hat{z}$; $U_{x,y} = 0,\ R_{x,y} = 0.$ (iii) U_z free; (iv) $U_z = 0.$
c	Cylinder waterbomb	No platens; global rigid body motions suppressed.	Reference constraint to remove rigid motion along or around $\hat{z}$: $U_z = 0,\ R_z = 0.$	Actuation on the coloured sectors (i, ii, iii): outward radial displacement $\Delta r > 0$; elsewhere free.
d	Waterbomb wheel (large)	Ends kinematically tied to rigid platens.	Bottom platen fixed: $U_{x,y,z} = 0,\ R_{x,y,z} = 0.$	Compression $U_z = -u$; $U_{x,y} = 0,\ R_{x,y,z} = 0.$
e	Curved-crease strip	No rigid platens are used.	Two bottom long edges: $U_z = 0$; in-plane motion and rotations are free.	Along the top crease, impose $U_z = -u$; in-plane motion and rotation are free.
f	Curved-crease tube	Rims interact with platens only in z.	Bottom rim: $U_z = 0$; in-plane motion and rotations are free.	Top rim: $U_z = -u$; in-plane motion and rotations are free.

Table S3. Boundary conditions for Fig. 3a–d.

Panel	Specimen	Lower boundary	Upper boundary
a	Case I	$U_z = 0$.	Displacement-controlled compression in z: $U_z = -u$ with freedom in x and y.
b	Case II	$U_z = 0$.	Displacement-controlled stretching in z: $U_z = +u$ with freedom in x and y.
c	Case III	Fully fixed: $U_x = U_y = U_z = 0$.	Compression along the edges as indicated by the arrows; $U_x = -u,\ U_y = U_z = 0$.
d	Case IV	BC1: $U_z = 0$. BC2: $U_x = 0$.	BC1: $U_z = -u$. BC2: $U_x = +u$.

Table S4. Default tolerances and quick tuning guide for the compatibility test.

**Symbol**	**Meaning**	**Default range**
η_m	Max membrane-energy fraction (pre-screen)	1–5%
η_s	Max shear-energy fraction (pre-screen)	0.5–2%
`tol`	Max bending-energy misfit (fine check)	0.2

Table S5. Effective line-spring stiffness per unit crease length used in Fig. 4. Units: $\mathrm{N\,rad^{-1}}$.

Case	k_{up}	k_{mid}	k_{bottom}
Fig. 4c (programmable stiffness)	1.3	0.2	0.2
Fig. 4f (multistable metamaterial)	2.5	0.2	0.2
Fig. 4h (multi-plateau metamaterial)	1.3	0.2	0.2

Table S6. Thickness pairs for Fig. 4c. Units: mm.

Design	t_{L1}	t_{L2}
Positive stiffness	2.10	1.15
Near-zero stiffness	1.75	1.15
Negative stiffness	1.55	1.15

Table S7. Parameters for Fig. 4f. Thickness in mm; angles in degrees.

Layer (top→bottom)	t_{L1}	t_{L2}	$\theta_{0,L1}$	$\theta_{0,L2}$
1st	2.41	0.52	60°	42°
2nd	2.59	1.31	60°	48°
3rd	2.90	1.71	60°	45°

Table S8. Parameters for Fig. 4h. Thickness in mm; angles in degrees.

Layer (top→bottom)	t_{L1}	t_{L2}	$\theta_{0,L1}$	$\theta_{0,L2}$
1st	1.69	0.94	60°	45°
2nd	1.72	1.42	60°	50°
3rd	2.06	1.71	60°	50°

Captions for Movies S1 to S6

Caption for Movie S1. Geometry–mechanics compatibility test. Step-by-step illustration of the geometry–mechanics compatibility test.

Caption for Movie S2. Multistable metamaterial under quasi-static compression. Quasi-static compression test of the multistable metamaterial inverse-designed by Mode II for a prescribed mechanical response, together with the corresponding load–displacement curve.

Caption for Movie S3. Multi-plateau metamaterial under quasi-static compression. Quasi-static compression test of the multi-plateau metamaterial inverse-designed by Mode II for a prescribed mechanical response, together with the corresponding load–displacement curve.

Caption for Movie S4. Target-shape folding process of curved-crease origami. Folding process of the curved-crease origami structure inverse-designed by Mode II to achieve a target shape.

Caption for Movie S5. Origami manipulators with programmable kinematic primitives. Origami manipulators inverse-designed by Mode II to realise the basic kinematic primitives–extension, bending, and twisting–as well as their tunable dynamic responses enabled by different fabrication routes.

Caption for Movie S6. Representative grasping tasks of inverse-designed origami manipulators. Representative grasping tasks performed by origami manipulators inverse-designed by Mode II for complex motion paths, showing two selected path designs from the broader programmable design space, together with demonstrations of a UAV-mounted manipulator for open-space grasping and grasping inside a pipe-like confined environment.

Captions for Data S1 to S2

Caption for Data S1. Code for the meshless high-fidelity analysis (Mode I). Code implementing the meshless high-fidelity analysis (Mode I), which serves as the reference model for non-rigid origami and provides the basis for the geometry–mechanics compatibility test.

Caption for Data S2. Code for the reduced-order model and inverse design (Mode II). Code implementing the reduced-order Mode II derived from the geometry–mechanics compatibility test, together with its use in inverse design.
